# Particle‐Associated Bacterioplankton Communities Across the Red Sea

**DOI:** 10.1111/1462-2920.70075

**Published:** 2025-03-17

**Authors:** Larissa Frühe, Shannon G. Klein, Carlos Angulo‐Preckler, Anastasiia Martynova, Taiba Alamoudi, Jacqueline V. Alva García, Silvia Arossa, Jessica Breavington, Sofia Frappi, Elisa Laiolo, Kah Kheng Lim, Anieka J. Parry, Eleonora Re, Diego E. Rivera Rosas, Mattie Rodrigue, Alexandra Steckbauer, Vincent A. Pieribone, Mohammad A. Qurban, Carlos M. Duarte

**Affiliations:** ^1^ Marine Science Program, Biological and Environmental Science and Engineering Division (BESE) King Abdullah University of Science and Technology (KAUST) Thuwal Kingdom of Saudi Arabia; ^2^ OceanX New York New York USA; ^3^ National Center for Wildlife (NCW) Riyadh Kingdom of Saudi Arabia

**Keywords:** bacteria, environmental genomics, microbial communities, microbial ecology

## Abstract

Pelagic particle‐associated bacterioplankton play crucial roles in marine ecosystems, influencing biogeochemical cycling and ecosystem functioning. However, their diversity, composition, and dynamics remain poorly understood, particularly in unique environments such as the Red Sea. In this study, we employed eDNA metabarcoding to comprehensively characterise bacterioplankton communities associated with pelagic particles in a three‐dimensional assessment spanning depths from the surface to a depth of 2300 m along the full length of the eastern Red Sea within the exclusive economic zone of the Kingdom of Saudi Arabia. Our results reveal a diverse assemblage of taxa, with Pseudomonadota, Cyanobacteriota, and Planctomycetota being the dominant phyla. We identified pronounced spatial variability in community composition among five major Red Sea geographical regions, with a third of all amplicon sequence variants being unique to the Southern Red Sea in contrast to a relatively homogenous distribution along the water column depth gradient. Our findings contribute to a deeper understanding of microbial ecology in the Red Sea and provide valuable insights into the factors governing pelagic particle‐associated bacterioplankton communities in this basin.

## Graphical abstract

This study provides a comprehensive exploration of pelagic particle‐associated bacterioplankton in the Red Sea, revealing distinct biogeographical patterns and depth‐related community structures. Using eDNA metabarcoding, we highlight the dominance of Pseudomonadota, Cyanobacteriota, and Planctomycetota, with notable regional variability. Our findings enhance the understanding of microbial dynamics in this unique marine system, shedding light on the ecological factors shaping bacterioplankton diversity and distribution across spatial and vertical gradients.

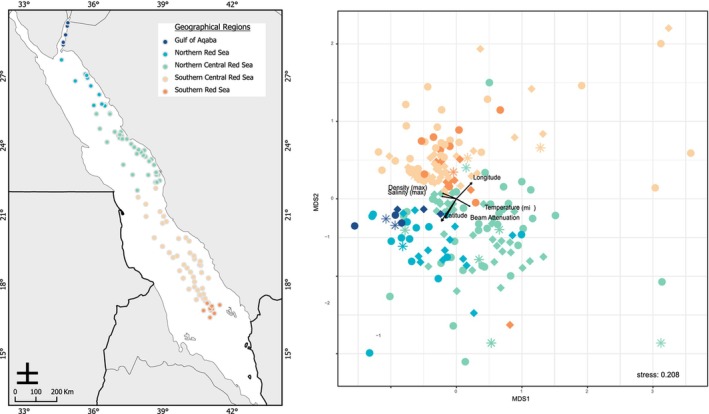

## 1 Introduction

Bacterioplankton are biodiverse and form a complex network of diverse metabolisms that ultimately impact nutrient and carbon cycling in pelagic ecosystems (Azam and Malfatti 2007; Falkowski et al. 2008; Grossart et al. 2020). Within bacterioplankton communities, heterotrophic bacteria constitute the majority of ocean biomass (Cotner and Biddanda 2002; Kirchman 2008), processing half of the organic carbon produced in the marine environment (Ducklow 1999). Furthermore, photosynthetic cyanobacteria, such as *Prochlorococcus* and *Synechococcus*, account for ~50% of the ocean's primary production (Pomeroy et al. 2007) and play a major role in the carbon and nutrient cycles within bacterioplankton‐based oligotrophic ecosystems (Armengol et al. 2019; Flombaum et al. 2013; Fuhrman et al. 2015; Arrigo 2005).

The composition of bacterioplankton communities varies within and across oceanic basins, depending on environmental forcing, such as depth (Sebastián et al. 2021; Sunagawa et al. 2015), temperature (Baldwin et al. 2005; Fuhrman et al. 2008), light and nutrient availability (Abell and Bowman 2005), and other water mass characteristics (Galand et al. 2010; Teira et al. 2006). This variability is also driven by dispersal limitations, which differ between surface and deeper waters, as currents, fronts, and water masses act as physical barriers separating communities (Baltar and Aristegui 2017; Morales et al. 2018; Raes et al. 2018).

Marine microbes associated with particles (PA microbes) play a crucial role in the degradation of particulate organic matter (POM). This is partly due to their capacity to produce extracellular enzymes, facilitating the hydrolysis of organic carbon (OC) and dissolved organic matter (DOM) (Arnosti 2011; Arnosti et al. 2021). A substantial proportion of the OC generated in the epipelagic zone is exported into the deep ocean via sinking particles (Ducklow et al. 2001), thus contributing to a globally significant carbon flux. This flux sustains microbial communities in the comparably nutrient‐depleted bathypelagic realm with fewer available nutrients (Arístegui et al. 2009). PA communities often exhibit higher metabolic activity and versatility (D'ambrosio et al. 2014; Eloe et al. 2011; Garneau et al. 2009) and greater phylogenetic diversity (Eloe et al. 2011; Ortega‐Retuerta et al. 2013) compared to their free‐living counterparts.

Rising ocean temperatures are expected to increase heterotrophic bacterioplankton abundance in marine ecosystems (Morán et al. 2015; Sarmento et al. 2010). The Red Sea provides an exceptional model system for studying the potential impact of ocean warming on marine ecosystems due to its abnormally warm and more uniformly distributed temperatures throughout the water column. Temperatures range from 35°C in surface waters to a minimum of 21°C in deeper waters, even at the basin's deepest point (Yao and Hoteit 2018). In addition, the Red Sea is also highly saline (> 40 psu) and hosts a well‐defined oxygen‐depleted (< 30 μmol kg^−1^) layer at mid‐depths (Naqvi 2021; Sofianos and Johns 2007; Hubert‐Huard et al. 2024). Previous works on the central Red Sea's epi‐ and mesopelagic coastal waters (Calleja et al. 2018; García et al. 2018) have suggested that abundances of heterotrophic bacteria (~5 × 10^5^ cells mL^−1^) may be notably lower than estimated values—from 10^6^ (Gasol and Kirchman 2018) to 10^29^ (Whitman et al. 1998)—in the world's oceans, attributed to the Red Sea's unique condition and oligotrophic nature. Despite its significance, research efforts in the Red Sea have predominately focused on specific areas such as the Gulf of Aden or Gulf of Aqaba regions (Grossart and Simon 2002), while most global expeditions like Galathea 3 (www.galathea3.dk/uk) or the Malaspina Expedition (Duarte 2015) have excluded the Red Sea entirely.

Investigating the dynamics of PA bacterioplankton in the Red Sea, including environmentally driven community shifts across the water column and latitudinal dispersal, holds the potential to elucidate the functioning of bacterioplankton communities in a warming ocean. In this study, we present a comprehensive description of Red Sea PA bacterioplankton communities based on an extensive dataset of samples collected during the Red Sea Decade Expedition (RSDE 2022) (www.rsde.ncw.gov.sa), which surveyed Saudi Arabia's Eastern Red Sea between February and June 2022. We collected 209 samples from the epipelagic, mesopelagic, and bathypelagic layers using an innovative Bottle Net system across the full latitudinal gradient of the Red Sea (108 distinct stations) targeting particle‐associated bacteria > 20 μm. Leveraging advanced molecular techniques, we extracted and amplified eDNA, targeting the hypervariable V3V4 region of the 16S SSU rRNA gene for an in‐depth assessment of bacterioplankton communities. Full‐depth CTD deployments enabled the characterisation of environmental conditions at each site. Our analyses yield insights into particle‐associated bacterioplankton diversity, distribution, and ecological roles, enhancing understanding of microbial community dynamics and biogeographical patterns in the Eastern Red Sea.

## 2 Materials and Methods

**FIGURE 1:**
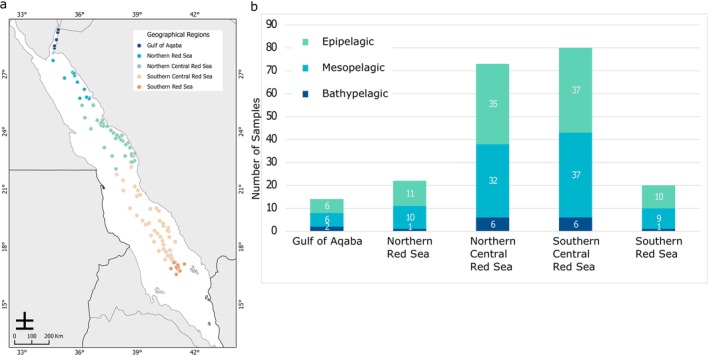
Locations of water column samples taken during the Red Sea Decade Expedition. (a) Overview map of the Red Sea with sampling locations (*n* = 108) (b) Composition of the 209 samples taken and their attribution to the five geographical regions (Gulf of Aqaba, Northern Red Sea, Northern Central Red Sea, Southern Central Red Sea, Southern Red Sea) and water column layers (Epipelagic, Mesopelagic, Bathypelagic). GPS coordinates and environmental parameters can be found in File S1.

The Red Sea Decade Expedition 2022 (RSDE) sailed the eastern coast of the Red Sea aboard R/V OceanXplorer and R/V Al Azizi between 4 February and 16 June 2022, sampling primarily during Spring (March–May). The expedition was divided into four Legs, each covering approximately one‐quarter of the eastern Red Sea (Figure 1). Within each Leg, sampling was divided into two phases: a deep phase that targeted stations between 500 and > 2000 m depth (Phase I), followed by a shallow phase, which focused on stations ranging shallower than 500 m (Phase II). Departing from Jeddah (Saudi Arabia), the expedition's first Leg sampled between 19.5° and 22.5° latitude. In Leg 2, between March and April 2022, R/V OceanXplorer sailed southwards to explore the southern Red Sea within 16.5°–19.5° latitude. In late April 2022, the expedition travelled northward to sample between 22.5° and 25.5° latitude during Leg 3. Lastly, the final Leg of the expedition commenced in mid‐June 2022, targeting the northern Red Sea north of 25.5° latitude, encompassing the Gulf of Aqaba.

For the analyses, the Red Sea was divided into five distinct regions: the Gulf of Aqaba (AQB), Northern Red Sea (NRS), Northern Central Red Sea (NCRS), Southern Central Red Sea (SCRS), and Southern Red Sea (SRS) based on the findings by (Raitsos et al. 2013), closely aligning with the expedition's split in the four distinct Legs (Figure 1a). They analysed satellite‐derived chlorophyll‐a concentrations to investigate phytoplankton dynamics in the Red Sea, distinguishing the four different areas based on their productivity and trophic state, with each exhibiting unique seasonal patterns (Raitsos et al. 2013).

### 2.1 Sampling

Sampling was carried out using the Bottle‐Net (Agustí et al. 2015), a patented oceanographic device comprising a 20 μm mesh plankton net housed in a cylindrical PVC pipe that integrates plankton communities along discrete depth layers (Agustí et al. 2015). The device features a net mouth positioned at the top and a hermetically sealed entrance, similar to Niskin bottles, and is mounted on a standard rosette sampler using a shooter mechanism to open and close the lids of the Bottle‐Net. Upon activating the rosette's mechanism to open the Bottle‐Net at a specified depth, sampling occurs during the ascent. This continues until the system triggers a second position to securely close the lid. Each Bottle‐Net collects a single, integrated plankton sample per deployment between the depth of opening and the depth of closure of the lid. Because of the mesh size of the plankton net, the fraction of the bacterioplankton community sampled was that is, > 20 μm particle‐attached fraction, which is the most versatile and active metabolically (D'ambrosio et al. 2014; Eloe et al. 2011; Garneau et al. 2009).

Sampling stations (*n* = 108) (coordinates can be found in File S1) were strategically divided into two or three standard vertical water layers based on depth to ensure comprehensive coverage of the water column. The layers sampled using distinct bottle‐nets mounted in the rosette sampling system encompass the epipelagic (ranging from 2–3 to 180 m depth), the mesopelagic layer (spanning from 180 to 1000 m depth), and the bathypelagic layer (extending from 1000 m to the sea floor), with each of the bottle nets opened at the bottom depth and closed at the upper depth of the layer sampled. This depth‐stratified sampling approach enabled systematic sampling across different depth strata, facilitating a comprehensive assessment of the marine ecosystem compared with discrete sampling with standard Niskin bottles, which may miss layers supporting dense particle layers. In total, 209 samples were collected, of which 99 were sampled in the epipelagic layer, 94 in the mesopelagic layer, and 16 in the bathypelagic layer (Figure 1b). The limited number of samples from the bathypelagic layer was constrained by the bathymetry of the expedition's operative sites.

Once onboard, the nets inside the bottle nets were gently rinsed with filtered seawater to collect all particles from the mesh in the net bucket. Then, the content of the sample container (~500 mL) was transferred to a plankton splitter and washed with ~150–200 mL 70% Ethanol (EtOH) and stored at +4°C for < 24 h before filtering.

### 2.2 Sample Processing

The contents of the bottle net in EtOH were then filtered through a 0.45 μm pore‐size filter (Pall Life Sciences, Port Washington, NY, USA) using a portable peristaltic pump (Sentino microbiology pump, Pall Life Sciences) set to a flow rate of 20 mL/100 s. All filters utilised in the process were sterile, 47 mm in diameter, and composed of mixed nitrocellulose esters. Sterile Filters were carefully placed in prelabelled sterile Eppendorf tubes (ThermoFisher Scientific, Waltham, MA, United States) and stored at −80°C until further processing. To minimise the risk of contamination, filter holders were replaced between each sample, and the system was thoroughly sterilised by employing a 10% commercial bleach solution and 70% EtOH rinse followed by MilliQ (Burlington, MA, USA) Ultrapure deionised water.

DNA from the filters was extracted using the DNeasy PowerWater DNA Isolation Kit (Qiagen, Hilden, Germany). The kit was used following the manufacturer's recommended protocol. The isolated DNA was eluted in a final volume of 100 μL in elution buffer and stored at −20°C. Extracted DNA was quantified using the Qubit 4.0 Fluorometer (ThermoFisher Scientific, Waltham, MA, United States) with the Qubit 1X dsDNA HS Assay Kit (ThermoFisher Scientific) and then amplified targeting the hypervariable V3V4 region of the 16S SSU rRNA (~450 bp length) gene using the 314F/805R primer set (Herlemann et al. 2011).

PCR was conducted in triplicate with the following parameters: Initial denaturation at 98°C for 2 min, then 30 cycles of 98°C for 20 s, 54°C for 20 s, and 72°C for 15 s, closing with a final elongation at 72°C for 2 min. The PCR reaction mix consisted of 5 μL KAPA 2× Master Mix (Roche), 3.25 μL of PCR‐grade water, 1 μL of 5 mM forward primer, 1 μL of 5 mM reverse primer, and 0.25 μL of 20 mg/mL BSA (20 mg/mL, ThermoFisher Scientific). Successful amplification of the target gene was verified using gel electrophoresis. Final PCR products were pooled, cleaned using AMPure XP Beads (Beckman Coulter, Brea, CA, USA), and then indexed using the Illumina Inc. Nextera XT kit (Illumina, San Diego, CA, USA). Amplicon libraries were normalised and sequenced on an Illumina NovaSeq6000 platform (2 × 250 bp) at Bioscience Core Lab at King Abdullah University of Science and Technology.

### 2.3 Bioinformatic Pipeline

Raw sequence data were demultiplexed, followed by primer removal using cutadapt v.4.4 (Martin 2011) with Python 3.10.10. The trimmed paired‐end sequences were then quality filtered (maxEE = 1, truncLen = 230) and clustered in amplicon sequence variants (ASV) using the Divisive Amplicon Denoising Algorithm (Rosen et al. 2012) from dada2 v.1.24.0 in R (Callahan et al. 2016) and subsequently merged with a minimum overlap of 20 bp and allowed mismatch of 2. Sequences with a basepair length of > 460 and < 440 were discarded, and chimeras were removed using the removeBimera function. Sequences were assigned to their respective taxonomy using the assignTaxonomy function in dada2, using the RDP naive Bayesian classifier method (Wang et al. 2007) and the SILVA v.138.1 database as reference (Quast et al. 2012). ASVs identified as contaminants by the decontam package v.1.24.0 (Davis et al. 2018) were removed from the ASV‐to‐sample matrix right after. Further, all ASVs with reads < 10 were removed from the dataset to reduce noise (Bokulich et al. 2013) as well as ASVs with non‐target taxonomy (e.g., assignment of “Eukaryotes” on phylum level instead of “Bacteria”, mitochondria or chloroplasts). To acknowledge the constant change in taxonomic nomenclature for bacteria, phylum names retrieved from the SILVA v.1.138.1 database were changed in accordance with the most recent suggestions by LPSN (Parte et al. 2020). A tracking of total read numbers can be found in File S2. A single phyloseq v.1.40. object (McMurdie and Holmes 2013) was created encompassing all environmental data, taxonomic assignment, and read counts.

### 2.4 Environmental Data

The SeaBird 911 plus CTD profiled the water column at all 108 stations. In addition, sensors equipped on the CTD measured temperature (°C), salinity (psu), pressure, beam attenuation (proxy of turbidity with optical scattering at 700 nm wavelength), dissolved O_2_ (*d*O_2_), fluorescence (proxy measure of optical scattering 470/695 nm indicating chlorophyll a), and seawater density. Before the expedition, all CTD sensors underwent calibration at the SeaBird laboratory. In April 2022, the bottle nets were replaced by 12 L Niskins on the Rosette sampling system, enabling the collection of discrete seawater samples to validate the accuracy of *d*O_2_ concentrations measured in the CTD profiles. For this purpose, two sites were selected, characterised by well‐oxygenated surface layers and deoxygenated bottom layers (ranging from 179 to < 2 μmol O_2_ kg^−1^). Although no bottle nets were deployed at these sites and water samples from Niskin bottles were not included in the analysis presented here, they provided an opportunity to test the CTD accuracy over a wide range of oxygen concentrations. *d*O_2_ concentrations in the discrete water samples were determined using an automated Winkler titration method (Carpenter 1965) and were used to verify CTD measurements. To correlate the environmental data measured by the CTD with the eDNA metabarcoding samples from the bottle nets, we extracted the following statistics from each layer (epi‐, meso‐, and bathypelagic) of the CTD profiles: mean, median, max, and min (File S1). The ‘oce’ package v.1.8–2 in R was used to extract the data and calculate summary statistics.

We examined multicollinearity among the 24 independent environmental variables using Variance Inflation Factors (VIFs), which were calculated using the ‘car’ v.3.1–2 package in R. If a factor was collinear with another (VIF > 5), the factor with the highest VIF value was removed, and the analysis was rerun in a stepwise manner until the remaining independent factors achieved VIF values < 5 (Files S3 and S4).

### 2.5 Data Analysis

The bacteria dataset was standardised to even sampling depth using the median sequence read count per sample (*n* = 618,747). Alpha diversity metrics were calculated using the ‘phyloseq’ package v.1.40 (File S5). We used linear models to test the effect of geographical region (with five levels: AQB, NRS, NCRS, SCRS, and SRS), layer (with three categories: epi‐, meso‐, and bathypelagic), and their interaction on the alpha diversity metrics: observed richness, Chao1, ACE, Shannon, Simpson, and Fisher (File S6). Prior to analyses, we used standardised residual plots and Q‐Q plots to check for normality and homoscedasticity and, if required, the data were transformed (File S7). If any terms were significant, estimated marginal means were used to determine which means differed.

Beta‐diversity analyses were conducted by calculating Bray‐Curtis (BC) dissimilarity values, which were then used for nonmetric multidimensional scaling (NMDS) using the metaMDS function in ‘vegan’ v.2.6–4 (Oksanen et al. 2007). Environmental parameters from CTD sensors were fitted against the NMDS using the envfit function (File S8) and significant (*p* < 0.05) parameters were depicted in the plot. To evaluate variations in bacterioplankton community composition, we also employed Analysis of Similarities (ANOSIM) using Bray‐Curtis distance with the anosim function in vegan v.2.6–4 (File S9). Venn diagrams were calculated and designed using the ‘eulerr’ v.7.02 package.

All processing and analyses for sequencing and water chemistry data were conducted in R v.4.2.2 using RStudio v.2022.12.0 + 353. A comprehensive list of all code, session information, and parameters for bioinformatic processing, statistical analysis, data curation, and visualisation can be found online at https://github.com/lexscience/Fruehe2024‐Bacterioplankton. Sequence data is available under bioproject number PRJNA980126 in the NCBI Sequence Read Archive (SRA).

## 3 Results

### 3.1 Environmental Profiling

**FIGURE 2:**
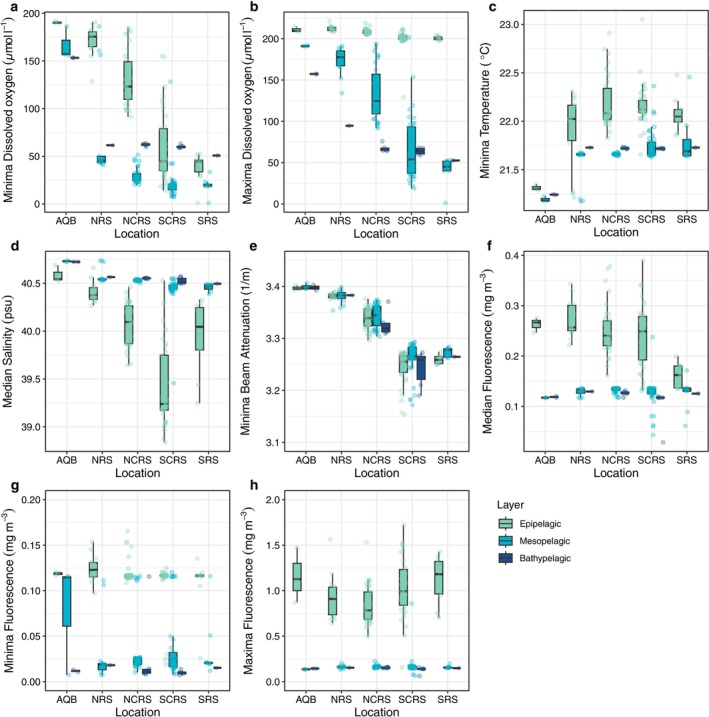
Environmental parameters characterising the epipelagic, mesopelagic and bathypelagic layers within geographical regions of the Red Sea. (a) Minima dissolved oxygen (μmol L^−1^) (b) Maxima dissolved oxygen (μmol L^−1^) (c) Minima temperature (°C), (d) Median salinity (psu), (e) Minima beam attenuation (1/m), (f) Median fluorescence (mg m^−3^), (g) Minima fluorescence (mg m^−3^), and (h) Maxima fluorescence (mg m^−3^). Tukey's boxplots depict the distribution of values, with raw data overlaid for clarity. Minima and maxima values have been calculated based on all measured values within one layer.

Eight of the initial 24 environmental variables were retained for further analysis after VIF analysis: minimum temperature, minimum dissolved oxygen, maximum dissolved oxygen, median salinity, minimum beam attenuation, as well as median, maximum, and minimum fluorescence. Dissolved oxygen exhibited a pronounced gradient with latitude, with higher values observed in the northern compared to the central and southern Red Sea (Figure 2a,b). Maximum oxygen levels were lower in the bathypelagic layers, while minimum oxygen levels in these layers exceeded those in the mesopelagic layers across all regions. These findings align with the perennial oxygen‐depleted layer present in the southern and central Red Sea, where oxygen levels in the epipelagic and mesopelagic layers drop below 30 μmol kg^−1^ (Naqvi 2021). However, there was a reprieve from oxygen depletion in the deeper bathypelagic zones, suggesting a departure from the oxygen‐depleted layer previously observed at mid‐depths. In the Gulf of Aqaba, median temperature was substantially lower than that of the main Red Sea water body (Figure 2c), whereas median practical salinity peaked in this region (Figure 2d) with overall lower salinity in the epipelagic layer throughout all geographic regions. Beam attenuation showed similar values between the layers for all geographical regions, with a southward decrease in attenuation (Figure 2e). Median, minimum, and maximum fluorescence showed higher values in the sunlit epipelagic layer (Figure 2f–h) and similar measurements for meso‐ and bathypelagic layers. For maximum fluorescence, a dent can be observed in the Northern and Northern Central Red Sea (Figure 2h).

### 3.2 Metabarcoding Data

Illumina NovaSeq6000 sequencing yielded a total of 391,682,180 raw reads, of which an average of 70% were kept throughout all steps of quality filtering and chimera removal (File S2). After noise reduction and taxonomic target clearing, the final table consisted of 161,078,928 high‐quality reads distributed in 79,739 unique ASVs, of which all could be assigned down to family level. Assigned to genus level were 120,326,301 reads in 52,120 ASVs and 878 ASVs with 13,384,763 reads were assigned down to species level.

### 3.3 Diversity of Bacterioplankton Communities

**FIGURE 3:**
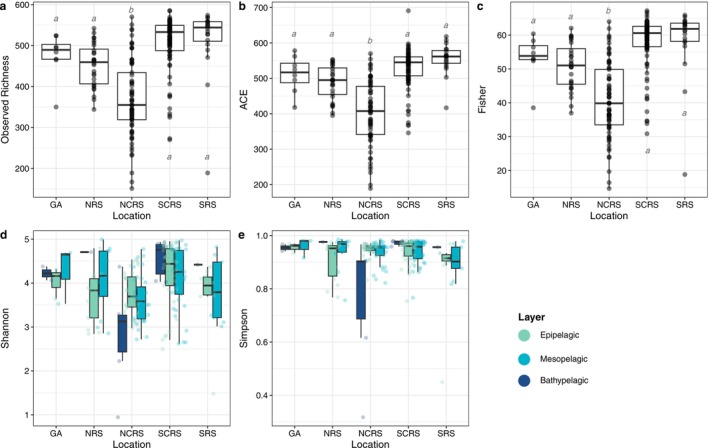
Tukey boxplots of alpha diversity measures of all geographical regions within the Red Sea along all water column layers. Geographical region abbreviations as follows: SRS, Southern Red Sea; SCRS, Southern Central Red Sea; NCRS, Northern Central Red Sea; NRS, Northern Red Sea; AQB, Gulf of Aqaba. For panels (a–c), layers are combined, as they have no significant effect on alpha diversity measures (File S7), whereas Layer × Location are depicted for panels (d) and (e). Statistical comparison and significance values for all measures can be found in File S7.

Geographical region and water column layer had no detectable effect on Chao1, whereas Observed richness, ACE, and Fisher index were significantly affected by geographical region according to linear models (see linear model results in File S7). There were significant interactions between layer and geographical region on Shannon and Simpson's index (File S7), translating to differences in layers depending on the geographical region. The observed richness as a measure of alpha diversity of the three distinct water column layers across the five geographical regions showed a unimodal pattern along the Red Sea. Post hoc analyses revealed that bacterioplankton community richness from the Gulf of Aqaba differed significantly from the Northern Central Red Sea but showed no significant differences to richness in the Northern Red Sea, Southern Central Red Sea or Southern Red Sea (Figure 3a, File S7). Main effects of location on Observed richnesss, ACE and Fisher indices were caused by lower values in the Northern Central Red Sea across all metrics relative to all other locations, regardless of water column layer. We detected significant interactions between location and layer for Shannon and Simpson indices, which resulted from differences between layers that depended on location. Specifically, post hoc analyses revealed that the North Central Red Sea had distinct differences in Shannon and Simpson values among the layers, whereas all other locations showed homogeneous values across layers (File S7). The observed richness showed no statistical differences between meso‐ and bathypelagic layers of the water column along all the Red Sea, with slightly higher values in the epipelagic layer, but no detected significant differences for the layers, resulting in geographical region being the main driver of observed richness. The same trends and significances were detected for Fisher's index (File S7).

Shannon diversity of distinct water layer communities was not significantly different for the Gulf of Aqaba, Northern Red Sea, Southern Central Red Sea, and Southern Red Sea, with only significant differences between the epipelagic and bathypelagic (*p* = 0.001) and mesopelagic and bathypelagic (*p* = 0.008) layers in the Northern Central Red Sea (Figure 3b). The same patterns can be observed with the Simpson index, showing the same significant differences between epipelagic and bathypelagic (*p* ≤ 0.001) and mesopelagic and bathypelagic (*p* ≤ 0.001) communities.

### 3.4 Prevalent Members of Bacterioplankton Community

**FIGURE 4:**
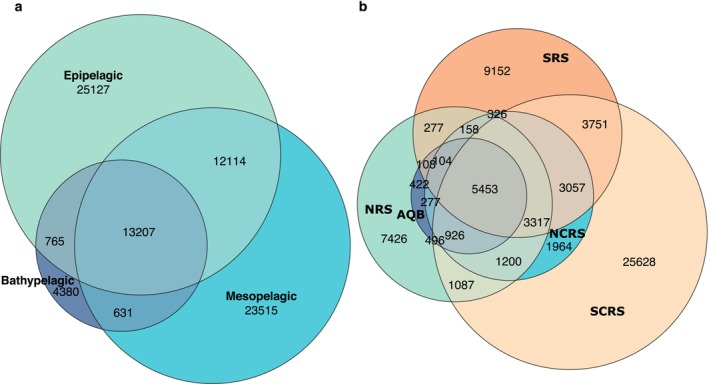
Venn diagrams with numbers of shared and unique ASVs per each Layer (a) and geographical region (b). Geographical region abbreviations as follows: Southern Red Sea (SRS), Southern Central Red Sea (SCRS), Northern Central Red Sea (NCRS), Northern Red Sea (NRS), and the Gulf of Aqaba (AQB).

Out of all 79,739 ASVs in this study, 30% are exclusively occurring in either the mesopelagic or epipelagic layer, and 5% are unique to the bathypelagic, whereas ASVs occurring ubiquitously through the water column make up 17% of the dataset (Figure 4a). The mesopelagic layer shares more ASVs with the epipelagic (*n* = 12,114, 15%) than with the bathypelagic layer (*n* = 631, 1%). The southern part of the central Red Sea harbours the most unshared ASVs in this region (32%) followed by the Southern Red Sea with 11.5% and the Northern Red Sea with 9% exclusive ASVs, whereas the Gulf of Aqaba and the Northern Central Red Sea share the majority of their ASVs with neighbouring areas (Figure 4b). Ubiquitously distributed within all geographical regions of the Red Sea are 7% of the ASVs (*n* = 5453). The smallest overlap can be found between the Gulf of Aqaba, Northern Central Red Sea, and Southern Red Sea, which only have 61 ASVs in common.

### 3.5 Taxonomic Composition of Bacterioplankton Communities

**FIGURE 5:**
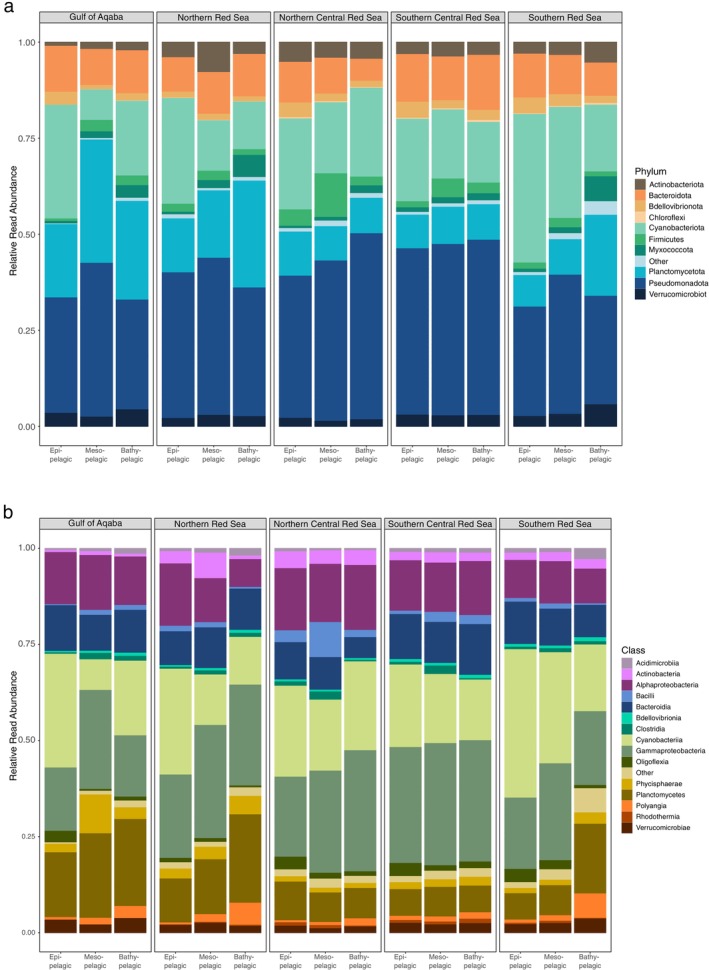
Relative read abundance by taxonomic assignment separated by geographical region. (a) Phylum‐level assignment displaying the 10 most abundant phyla in all water layers with low abundance phyla summarised in ‘Other’. (b) Class‐level assignments displaying the 15 most abundant classes in all water layers with low abundance classes summarised in ‘Other’. Geographical region abbreviations as follows: Southern Red Sea (SRS), Southern Central Red Sea (SCRS), Northern Central Red Sea (NCRS), Northern Red Sea (NRS), and the Gulf of Aqaba (AQB).

The most abundant phylum is Pseudomonadota (former Proteobacteria), followed by Cyanobacteriota and Planctomycetota (Figure 5a). In addition, the most abundant classes are Gammaproteobacteria and Cyanophyceae (Figure 5b). Out of the five most abundant ASVs, three were assigned to Cyanobacteriota and one to Pseudomonadota and Planctomycetota each. The most abundant ASV with 10,704,091 reads was assigned to the genus *Cyanobium*, and the second most abundant was assigned to the genus *Synechococcus*, both part of the family Cyanobiaceae. Furthermore, the species *Trichodesmium IMS 101 thiebautii*, followed by *Alteromonas* sp., comprise the third and fourth most abundant ASVs. The fifth most abundant ASV was an unclassified member of the family Pirellulaceae. All numbers of ASV abundance can be accessed in the raw ASV‐to‐sample matrix provided on DRYAD.

Overall, taxonomic composition at the phylum level was quite homogenous between layers and regions of the Red Sea (Figure 5a). Nonetheless, an increase of Myxococcota and Planctomycetota with depth can be observed, as well as a small increase of Chloroflexota with depth in the southern regions of the Red Sea. The abundance of Bdellovibrionota, Bacteroidota, and Actinomycetota stays mostly constant, whereas Cyanobacteria decrease in abundance with depth. At the class level, an increase in abundance with depth can be observed for Acidimicrobiia, Bdellovibrionia, Clostridia, Planctomycetes, and Polyangia, whereas a decrease in relative abundance can be observed for Oligoflexia (Figure 5b). Desulfonauticaceae showed a clear distribution pattern with higher relative read abundances in surface waters, whereas relative Bacillaceae numbers increased with depth.

Actinobacteria had their lowest relative abundance in the Gulf of Aqaba, as well as the cyanobacterial genera *Dactylococcopsis* sp. and *Lyngbya* sp., with *Bradymonadaceae* and *Staphylococcus* being completely absent from samples from the Gulf of Aqaba. In contrast, Phycisphaerae were more prevalent in the northern than in the southern areas of the Red Sea. Notable is the pronounced peak in the relative abundance of the photosynthetic and N‐fixing cyanobacterial genus *Trichodesmium* sp. in the Northern and Northern Central Red Sea (File S10). The southern regions of the Red Sea harboured far more *Cyanobium* sp. compared to the northern areas (File S10). Acidimicrobia also preferred the southern regions of the Red Sea, contrary to Desulfovibrionaceae and Clostridia, which were not as abundant in the southern Red Sea. The centre of the Red Sea hosted richness in Bacilli and Rhodothermia, which exhibited higher relative abundances in the Northern and Southern Central Red Sea. Sulfate‐reducing bacteria (SRBs) exhibited a higher total abundance in the Southern Central and Northern Central Red Sea (File S11). Bacterial taxa adaptable to high salinity, like, for example, Halanaerobiaceae (Oren et al. 1984), showed a prevalence for the deep waters of the Gulf of Aqaba and the surface waters of the Northern Red Sea, while being absent from other bathypelagic samples from the whole dataset.

### 3.6 Vertical and Spatial Community Structure

**FIGURE 6:**
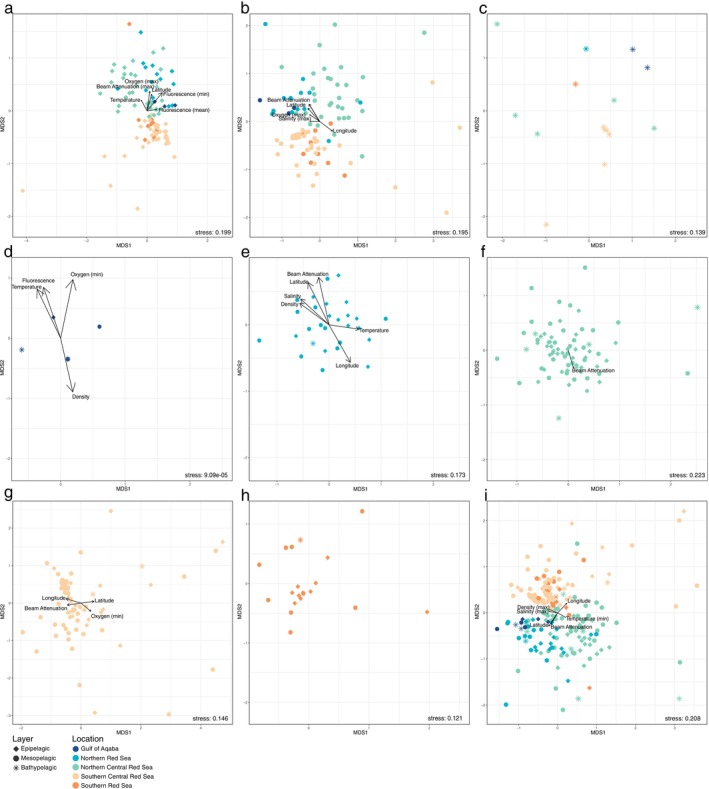
Bacterial community structure in the different water column layers of the Red Sea. (a–c) Nonmetric multidimensional ordinations based on Bray‐Curtis distance of bacterial communities from the (a) epipelagic, (b) mesopelagic and (c) bathypelagic water layer displaying distribution between the five geographical regions of the Red Sea distinguished by colour. Length of arrows indicates strength of correlation. Results of environmental fitting can be found in File S8. For increased readability, arrows stemming from the same variable (e.g., Temperature‐mean and Temperature‐median) pointing in the same direction were merged into a shared arrow. If only one variation of a parameter had significant values, it was noted in the figure (e.g., ‘Oxygen min.’). Only significantly correlating parameters are shown. (d–h) NMDS ordination of bacterial communities from (d) Gulf of Aqaba, (e) Northern Red Sea, (f) Northern Central Red Sea, (g) Southern Central Red Sea and (h) Southern Red Sea with shapes indicating different water column layers. (i) Ordination of the whole dataset with colours showcasing geographical region and shape defines water column layer.

There was a strong influence of environmental factors on distinct communities in the two uppermost layers of the water column (Figure 6a,b), whereas the bathypelagic realm is not significantly shaped by environmental parameters measured in this study (Figure 6c). Maximum oxygen (*p* = 0.001) and fluorescence (*p* = 0.006) are the main factors shaping the epipelagic community, whereas mesopelagic bacterial communities are mainly influenced by Latitude (*p* = 0.001) and beam attenuation (*p* = 0.001). A separation between the southern and northern realms of the Red Sea can be observed for the epipelagic layer (ANOSIM, Bonferroni‐corrected, *p* = 0.05; Figure 6a), with a non‐significant less pronounced shift in the mesopelagic (ANOSIM, Bonferroni‐corrected, *p* = 0.25; Figure 6b) and absent for the bathypelagic realm (ANOSIM, Bonferroni‐corrected, *p* = 0.81; Figure 6c). The shift between southern and northern communities occurs at around 21° N, separating the Red Sea into two distinct environments in the upper layers of the water column.

The main influences on bacterial communities in the Gulf of Aqaba are temperature (*p* = 0.003) and beam attenuation (*p* = 0.003) with a separation of communities by pelagic layer (ANOSIM, Bonferroni‐corrected, *p* = 0.01; Figure 6d). Major shaping by layer also occurred in the Northern Red Sea (ANOSIM, Bonferroni‐corrected, *p* = 0.008; Figure 6e), Northern Central Red Sea (ANOSIM, Bonferroni‐corrected, *p* = 0.0005; Figure 6f), and Southern Central Red Sea (ANOSIM, Bonferroni‐corrected, *p* = 0.09; Figure 6g), whereas the Southern Red Sea does not exhibit strict separation patterns for community origin within the water column (ANOSIM, Bonferroni‐corrected, *p* = 0.6; Figure 6h).

## 4 Discussion

Our results reveal a diverse PA bacterioplankton community in the Red Sea, comprising a total of 79,739 unique ASVs. Recent studies on Red Sea bacterioplankton revealed that surface waters are dominated by the autotrophic picoplankton cyanobacterial genus *Prochlorococcus* and the heterotrophic SAR11 group (Ngugi et al. 2012), which were also highly abundant in this study, confirming their prevalence in the Red Sea's epipelagic waters.

The high abundance of *Trichodesmium*—the fourth most abundant ASV in this study with pronounced abundance peaks in the two top layers in the Northern Central Red Sea—is not surprising as previous studies reported the occurrence of *Trichodesmium* in this region (Foster et al. 2009; Kürten et al. 2015). *T. erythraeum* was observed by Ehrenberg in the 19th century in the Red Sea for the first time (Ehrenberg 1830), and surface blooms, staining the surface of the ocean reddish‐brown, most likely naming the Red Sea, have been described and observed since as early as the 1970s (Kimor and Golandsky 1977). Moreover, *Trichodesmium* trichomes often bundle to form filaments of significant size visible with the naked eye, which are retained by the 20 μm mesh used in this study. While generally nitrogen‐fixing, the abundance of *Trichodesmium* is favoured by the combination of high water temperatures and low nutrient concentrations (Kürten et al. 2019). In northern regions, nutrients are intermittently accessible, with surface waters often exhibiting significant oligotrophy (Ngugi et al. 2012). Conversely, in the south, this deficiency is compensated for by the influx of nutrient‐rich water masses from the Indian Ocean via the Gulf of Aden (Sofianos et al. 2002) explaining the lower abundance of *Trichodesmium* in lower latitudes in the Red Sea. The generally observed high abundance of Cyanobacteriota in large particle fractions may result from various ecological interactions and particle‐associated behaviours. In addition to the presence of Trichodesmium, we hypothesise that other cyanobacterial taxa may contribute to these large particles due to surface‐derived organic matter aggregation, phytoplankton senescence, or by becoming trapped within that is, zooplankton faecal pellets or marine snow—organisms, and higher abundances of Cyanobacteria are not in situ growth in deeper layers of the water column. Cyanobacteria are known to produce extracellular polymeric substances (EPS), which could facilitate their adhesion to particles and enhance aggregation (Sun et al. 2020). While many cyanobacteria are small and primarily reside in the photic zone, colony‐forming genera such as *Trichodesmium* can aggregate into large particles that facilitate their sinking. Additionally, *Trichodesmium*'s role in nitrogen fixation enhances primary production, potentially increasing the availability of organic matter for particle formation and export (Capone et al. 1997).

Additionally, the presence of Cyanobium as the most abundant ASV could be explained by the common cyanobacterial tolerance to a range of nutrient conditions and light availability, enabling it to thrive across different microenvironments within these aggregates due to their metabolic versatility (Stal 2007). *Prochlorococcus* and *Synechococcus* stand out as the predominant and most widely distributed phytoplankton taxa in the world's oceans (Flombaum et al. 2013) and previous studies have found *Prochlorococcus* and *Synechococcus* to be dominating taxa within the sunlit layers of the Red Sea (Ngugi et al. 2012), whereas they were present in uncommonly low numbers in recent studies in the central Red Sea (Ansari et al. 2022; Sabbagh et al. 2023) compared to those found elsewhere (Flombaum et al. 2013; DuRand et al. 2001). However, those studies correspond to the total bacterioplankton community, whereas our results pertain to the > 20 μm particle‐associated community. Yet, picoplankton of the genus *Synechococcus* were dominant in the particle‐associated community, consistent with observations that these cells often form aggregates that rapidly sink in the ocean (Agustí et al. 2015). Hence, the cyanobacterial community in this study was mostly comprised of *Cyanobium* sp., *Synechococcus* sp., and *Trichodesmium* sp. (File S9). Total read abundance of the five most abundant cyanobacteria was higher in epi‐ and mesopelagic layers across all regions of the Red Sea. In contrast, there were equal numbers of cyanobacterial reads in the epi‐ and bathypelagic layers of the Gulf of Aqaba (File S10). This observation may be attributed to the weak water column stratification in the Gulf of Aqaba (Genin et al. 1995; Felis and Rimbu 2010) and the convective mixing of water layers resulting from upwelling and eddies, predominantly observed from April to November (Badran et al. 2005; Chase et al. 2006). This timing aligns with the sampling period of this study, which was conducted in June.

In the global ocean, heterotrophic particle‐associated bacteria stem predominantly from the SAR11 clade. This group comprises over half of the surface microbial communities and about a quarter of those in the sub‐epipelagic zone, with an estimated global population of 2.4 × 10^28^ cells (Morris et al. 2002). SAR11 members are widely distributed, inhabiting diverse environments such as tropical and polar regions, as well as coastal, freshwater, and brackish waters (Logares et al. 2010; Brown et al. 2012). Previous studies based on cell counts suggest that heterotrophic bacterial abundances in central Red Sea epipelagic waters are lower than the global ocean average (Arístegui et al. 2009; Calleja et al. 2018; García et al. 2018; Silva et al. 2019), possibly explaining the relatively low numbers of sequences associated with the SAR11 clade, although in general, heterotrophic bacteria are not only composed of this clade but a multitude of taxa. Although SAR11 is typically characterised as a small, free‐living bacterium with a highly streamlined genome, its presence in particle‐associated fractions suggests potential entrapment within extracellular polymeric substances (EPS) or attachment to organic aggregates. Given SAR11's oligotrophic lifestyle, it is unlikely to actively colonise particles but may be incorporated into sinking material through interactions with EPS‐producing microbes or adsorption onto detrital surfaces.

We observed inconsistencies in the variation of certain diversity metrics (Shannon and Simpson) with depth, noting that these metrics generally declined with increasing depth, but this trend was location‐dependent. Although our analyses were designed to be robust against differences in sample size across layers, the limited power in the bathypelagic layer may still affect this finding. Shannon and Simpson metrics themselves are known to both measure diversity, with Shannon emphasising species diversity, considering both richness and evenness, and hence are more sensitive to rare species. On the other hand, the Simpson index gives more weight to dominant species, which could explain the inconsistencies with depth.

The increase of alpha‐diversity with increasing depth represents a notable phenomenon, supported by findings from analogous ecosystems, including the Antarctic (Luria et al. 2014) and the Mediterranean (Pommier et al. 2010). This trend is further evidenced by the higher Shannon values in the bathypelagic layer of the Northern Red Sea and both southern regions. The absence of a clear water column layer structuring has been observed before (Mestre et al. 2018), owing to the dispersal of surface taxa into deeper layers by sinking particles (Mestre et al. 2018; Cram et al. 2015), which were the focus of this study. These taxa are proposed to be able to initiate the establishment of communities in the profound ocean depths upon detachment from their carrier particles (Sohrin et al. 2011). Additionally, taxa frequently more abundant in the deeper layers have been found in sunlit surface waters (Mestre et al. 2018).

The apparent homogeneity in bacterioplankton community structure along the water column within each geographical location suggests that specialists within the microbial community (such as SRB, thermo‐ or halotolerant clades) are responsible for hidden community changes. Between the geographical locations, for example, the distinctiveness of bacterial communities in the Northern Central Red Sea relative to all other areas of the Red Sea, likely arises from multiple factors. A potential explanation for the uniqueness of samples from the northern geographical regions could be depth, as the samples from the two northern geographical groupings classified within the bathypelagic layer are almost 1000 m deeper than the bathypelagic samples from the Southern Red Sea. It has been proposed that increasing pressure with depth affects community composition of marine bacteria (Tamburini et al. 2013; Marietou and Bartlett 2014) and the decrease in the quantity of DOM at depth could affect deep‐sea heterotrophic communities (Jiao et al. 2014). Uneven sampling coverage between the five distinct geographical regions led to a representation of the bathypelagic layer of the Northern Red Sea and Southern Red Sea by only one sample each, possibly augmenting the observed geographical differences of PA bacterioplankton.

The vertical homogeneity of bacterioplankton communities when compared to other oceans where significant differences have been found across different depths (Li et al. 2023; Treusch et al. 2009; Yu et al. 2014) may further be explained by attenuated sorting of bacterioplankton species at depth due to lower temperatures in the Red Sea, where the coldest temperature at depth (2278 m) is 21.8°C (File S3). Additionally, the perennial oxygen‐depleted layer which has been observed in the Red Sea (Naqvi 2021) is no longer prevalent in the northern parts, most likely leading to a shift in bacterial community composition throughout the water column. The observed homogeneity could also be owed to the extensive mesopelagic layer, ranging from 200 to 1000 m, potentially masking small‐scale compartmentalisation and hence contributing to a more uniform dispersal of properties throughout the water column. The oxygen‐depleted layer prevails in the Southern and Southern Central Red Sea, with oxygen levels below 30 μmol kg^−1^ between 80 and 300 m water depth, but higher oxygen concentrations below that. The scarcity of oxygen in the southern realms of the Red Sea explains the higher abundance of anaerobic sulphate‐reducing bacteria (SRB) in those areas; however, their high occurrence could also be linked to the formation of oxygen‐deficient micro‐niches within aggregates of organic matter (File S10) (Bertagnolli and Stewart 2018).

While mountains or rivers are prominent physical barriers in terrestrial settings, the presence of physical barriers to dispersal in marine environments may be less apparent. However, even in the marine realm, physical barriers exist (Palumbi 1994). The Red Sea harbours a pronounced hydrographic contrast between the northern and southern regions, with opposing gradients of salinity (increasing northwards) and temperature (increasing southwards) (Figure 2a,b) (Trommer et al. 2009) with maximum surface temperatures of between 26°C (±1.1°C) in the north to 31.3°C (±1.1°C) in the south (Osman et al. 2018) and salinity increasing from 36 psu to 41 psu with increasing latitude (Ngugi et al. 2012). As future climate scenarios predict elevated water temperatures, significant changes in microbial community composition are anticipated. Rising temperatures are likely to increase stratification, which could affect the depth of the deep chlorophyll maximum (DCM), thereby altering the light regimes available to euphotic zone microbes (Cheng et al. 2020). This stratification may also hinder oxygen dispersion to deeper waters by creating a physical barrier, impacting deeper microbial communities (Bertagnolli and Stewart 2018; Keeling et al. 2010). Increased stratification might reduce the quantity of sinking particles that reach bottom waters, affecting the nutrient dynamics essential for deep‐sea ecosystems (Deuser et al. 1981). Rising temperatures may also affect oxygen availability by reducing the oxygen solubility in the water column and increasing organismal oxygen demand (Deutsch et al. 2015; Schmidtko et al. 2017). As temperature and oxygen availability are two of the key factors that drive the distribution of marine organisms, they are likely to induce changes in the distribution of bacterioplankton communities in the coming decades (Deutsch et al. 2020). The complex interactions between autotrophic and heterotrophic communities will be crucial in shaping the accumulation and turnover of organic matter in the oceans of the future, with implications for both carbon cycling and overall marine ecosystem health (Andersson et al. 2015; Zhou et al. 2018).

## Author Contributions

**Larissa Frühe:** conceptualization, data curation, formal analysis, visualization, writing – original draft, methodology, investigation, writing – review and editing, software, validation, project administration. **Shannon G. Klein:** data curation, formal analysis, visualization, writing – review and editing, investigation. **Carlos Angulo‐Preckler:** writing – review and editing, methodology. **Anastasiia Martynova:** visualization, writing – review and editing, data curation. **Taiba Alamoudi:** writing – review and editing, data curation. **Jacqueline V. Alva García:** data curation, writing – review and editing. **Silvia Arossa:** data curation, writing – review and editing. **Jessica Breavington:** data curation, writing – review and editing. **Sofia Frappi:** data curation, writing – review and editing. **Elisa Laiolo:** data curation, writing – review and editing. **Kah Kheng Lim:** data curation, writing – review and editing. **Anieka J. Parry:** data curation, writing – review and editing. **Eleonora Re:** data curation, writing – review and editing. **Diego E. Rivera Rosas:** data curation, writing – review and editing. **Mattie Rodrigue:** supervision, resources, writing – review and editing. **Alexandra Steckbauer:** data curation, writing – review and editing. **Vincent A. Pieribone:** resources, supervision, writing – review and editing. **Mohammad A. Qurban:** resources, funding acquisition, writing – review and editing. **Carlos M. Duarte:** conceptualization, methodology, writing – review and editing, funding acquisition, resources, project administration, supervision.

## Conflicts of Interest

The authors declare no conflicts of interest.

## Supporting information


**File S1.** Sample metadata with location of sites and environmental parameters.

**File S2.** Overview of sequencing reads through processing pipeline.

**File S3.** Variance inflation factors—collinearity analysis.

**File S4.** Variance inflation factors of environmental variables.

**File S5.** Alpha diversity metrics.

**File S6.** Linear models.

**File S7.** Significance testing of alpha diversity.

**File S8.** Environmental fitting within NMDS for environmental parameters.

**File S9.** ANOSIM.

**File S10.** Relative abundance plot of Cyanobacteria.

**File S11.** Relative abundance plot of SRBs.

## Data Availability

Raw sequencing data is available under PRJNA980126 in the NCBI Sequence Read Archive (SRA). Environmental data and supplementary information have been deposited in DRYAD https://doi.org/10.5061/dryad.vt4b8gv1k. Code is available under https://github.com/lexscience/Fruehe2024‐Bacterioplankton. All data for the manuscript is available on Dryad under https://doi.org/10.5061/dryad.vt4b8gv1k, as well as on GitHub under https://github.com/lexscience/Fruehe2024‐Bacterioplankton.
