# Supplementary material for: Particle‐Associated Bacterioplankton Communities Across the Red Sea
Source: Environ Microbiol. 2025 Mar 17;27(3):e70075. doi: 10.1111/1462-2920.70075 (PMC11914372; doi:10.1111/1462-2920.70075)
Supplement: Supplementary file 6 — File S6. Linear models. [file EMI-27-e70075-s009.docx]

| **Variable** | | **Observed richness** | **Chao1** | **ACE** | **Shannon** | **Simpson** | **Fisher** |
| --- | --- | --- | --- | --- | --- | --- | --- |
| Transformation | | Ln (x) | Ln (x) | Ln (x) | None required. | Sqrt (x+1) | Ln (x + *a* + 1) |
| Information criterion | | BIC = -19.50  AIC = -22.77 | BIC = 243.43  AIC = 240.17 | BIC= -93.37  AIC= -96.60 | BIC= 419.11  AIC= 415.85 | BIC=-828.22  AIC= -831.49 | BIC=-206.68  AIC= -209.95 |
| **Source of variation** | **Numerator *df*** | ***P*** | ***p*** | ***p*** | ***p*** | ***p*** | ***p*** |
| Layer | 2 | 0.563  F=0.577 | 0.533  F = 0.630 | 0.620  F=0.480 | 0.518  F=0.661 | 0.755  F=0.281 | 0.773  F=0.257 |
| Location | 4 | **< 0.001**  F=18.297 | 0.178  F = 1.591 | **<0.001**  F=18.226 | **<0.001**  F=9.923 | **<0.001**  F=5.611 | **<0.001**  F=22.317 |
| Layer × Location | 8 | 0.572  F=0.836 | 0.949  F =0.340 | 0.839  F=0.522 | **0.022**  F=2.304 | **<0.001**  F=4.314 | 0.326  F=1.159 |

**Supplementary Table 6:** Summary of results for Linear models testing the effect of layer, location, and their interaction on the dependent variables: observed richness, Chao1, ACE, Shannon, Simpson, and Fisher.

*Where, *a* is the most negative value in the dataset.
