# Supplementary material for: Particle‐Associated Bacterioplankton Communities Across the Red Sea
Source: Environ Microbiol. 2025 Mar 17;27(3):e70075. doi: 10.1111/1462-2920.70075 (PMC11914372; doi:10.1111/1462-2920.70075)
Supplement: Supplementary file 7 — File S7. Significance testing of alpha diversity. [file EMI-27-e70075-s010.docx]

**Supplementary Table 7a: Significant effects of Location on Observed Richness**

| **Estimates^a^** | | | | | | |
| --- | --- | --- | --- | --- | --- | --- |
| Location | Mean | Std. Error | df | 95% Confidence Interval | |  |
|  |  |  |  | Lower Bound | Upper Bound |  |
| Gulf of Aqaba | 6.140 | .076 | 194 | 5.991 | 6.289 |  |
| Northern Central Red Sea | 5.853 | .033 | 194 | 5.787 | 5.918 |  |
| Northern Red Sea | 6.119 | .075 | 194 | 5.971 | 6.267 |  |
| Southern Central Red Sea | 6.233 | .033 | 194 | 6.168 | 6.298 |  |
| Southern Red Sea | 6.237 | .077 | 194 | 6.085 | 6.389 |  |
|  | | | | | | |

| **Pairwise Comparisons^a^** | | | | | | | | | | | |
| --- | --- | --- | --- | --- | --- | --- | --- | --- | --- | --- | --- |
| (I) Location | | (J) Location | | Mean Difference (I-J) | | Std. Error | | df | Sig.^c^ | 95% Confidence Interval for Difference^c^ | |
|  |  |  |  |  |  |  |  |  |  | Lower Bound | Upper Bound |
| Gulf of Aqaba | | Northern Central Red Sea | | .288^*^ | | .083 | | 194 | .006 | .053 | .522 |
|  |  | Northern Red Sea | | .022 | | .106 | | 194 | 1.000 | -.281 | .324 |
|  |  | Southern Central Red Sea | | -.093 | | .082 | | 194 | 1.000 | -.327 | .141 |
|  |  | Southern Red Sea | | -.096 | | .108 | | 194 | 1.000 | -.403 | .210 |
| Northern Central Red Sea | | Gulf of Aqaba | | -.288^*^ | | .083 | | 194 | .006 | -.522 | -.053 |
|  |  | Northern Red Sea | | -.266^*^ | | .082 | | 194 | .014 | -.499 | -.033 |
|  |  | Southern Central Red Sea | | -.380^*^ | | .047 | | 194 | <.001 | -.513 | -.248 |
|  |  | Southern Red Sea | | -.384^*^ | | .084 | | 194 | <.001 | -.622 | -.146 |
| Northern Red Sea | | Gulf of Aqaba | | -.022 | | .106 | | 194 | 1.000 | -.324 | .281 |
|  |  | Northern Central Red Sea | | .266^*^ | | .082 | | 194 | .014 | .033 | .499 |
|  |  | Southern Central Red Sea | | -.114 | | .082 | | 194 | 1.000 | -.347 | .118 |
|  |  | Southern Red Sea | | -.118 | | .107 | | 194 | 1.000 | -.423 | .187 |
| Southern Central Red Sea | | Gulf of Aqaba | | .093 | | .082 | | 194 | 1.000 | -.141 | .327 |
|  |  | Northern Central Red Sea | | .380^*^ | | .047 | | 194 | <.001 | .248 | .513 |
|  |  | Northern Red Sea | | .114 | | .082 | | 194 | 1.000 | -.118 | .347 |
|  |  | Southern Red Sea | | -.004 | | .084 | | 194 | 1.000 | -.241 | .234 |
| Southern Red Sea | | Gulf of Aqaba | | .096 | | .108 | | 194 | 1.000 | -.210 | .403 |
|  |  | Northern Central Red Sea | | .384^*^ | | .084 | | 194 | <.001 | .146 | .622 |
|  |  | Northern Red Sea | | .118 | | .107 | | 194 | 1.000 | -.187 | .423 |
|  |  | Southern Central Red Sea | | .004 | | .084 | | 194 | 1.000 | -.234 | .241 |
| Based on estimated marginal means | | | | | | | | | | | |
| *. The mean difference is significant at the .05 level. | | | | | | | | | | | |
| a. Dependent Variable: Observed_richness_ln. | | | | | | | | | | | |
| c. Adjustment for multiple comparisons: Bonferroni. | | | | | | | | | | | |
| **Univariate Tests^a^** | | | | | | |  |  |  |  |  |
| Numerator df | Denominator df | | F | | Sig. | |  |  |  |  |  |
| 4 | 194.000 | | 18.297 | | <.001 | |  |  |  |  |  |
| The F tests the effect of Location. This test is based on the linearly independent pairwise comparisons among the estimated marginal means. | | | | | | |  |  |  |  |  |
| a. Dependent Variable: Observed_richness_ln. | | | | | | |  |  |  |  |  |

**Supplementary Table 7b: Significant effects of Location on ACE Index**

| **Estimates^a^** | | | | | | | | | | | |  |  |  |
| --- | --- | --- | --- | --- | --- | --- | --- | --- | --- | --- | --- | --- | --- | --- |
| Location | | Mean | Std. Error | | df | | 95% Confidence Interval | | | | |  |  |  |
|  |  |  |  |  |  |  | Lower Bound | | | Upper Bound | |  |  |  |
| Gulf of Aqaba | | 6.216 | .062 | | 186 | | 6.094 | | | 6.337 | |  |  |  |
| Northern Central Red Sea | | 5.958 | .029 | | 186 | | 5.900 | | | 6.016 | |  |  |  |
| Northern Red Sea | | 6.173 | .061 | | 186 | | 6.052 | | | 6.294 | |  |  |  |
| Southern Central Red Sea | | 6.275 | .027 | | 186 | | 6.222 | | | 6.328 | |  |  |  |
| Southern Red Sea | | 6.321 | .063 | | 186.000 | | 6.196 | | | 6.446 | |  |  |  |
|  | | | | | | | | | | | |  |  |  |
| **Pairwise Comparisons^a^** | | | | | | | | | | | | | | |
| (I) Location | (J) Location | | | Mean Difference (I-J) | | Std. Error | | df | Sig.^c^ | | 95% Confidence Interval for Difference^c^ | | |  |
|  |  |  |  |  |  |  |  |  |  |  | Lower Bound | | Upper Bound |  |
| Gulf of Aqaba | Northern Central Red Sea | | | .258^*^ | | .068 | | 186 | .002 | | .064 | | .452 |  |
|  | Northern Red Sea | | | .043 | | .087 | | 186 | 1.000 | | -.204 | | .289 |  |
|  | Southern Central Red Sea | | | -.059 | | .067 | | 186 | 1.000 | | -.250 | | .132 |  |
|  | Southern Red Sea | | | -.105 | | .088 | | 186.000 | 1.000 | | -.356 | | .146 |  |
| Northern Central Red Sea | Gulf of Aqaba | | | -.258^*^ | | .068 | | 186 | .002 | | -.452 | | -.064 |  |
|  | Northern Red Sea | | | -.215^*^ | | .068 | | 186 | .018 | | -.408 | | -.023 |  |
|  | Southern Central Red Sea | | | -.317^*^ | | .040 | | 186 | <.001 | | -.430 | | -.204 |  |
|  | Southern Red Sea | | | -.363^*^ | | .070 | | 186 | <.001 | | -.561 | | -.165 |  |
| Northern Red Sea | Gulf of Aqaba | | | -.043 | | .087 | | 186 | 1.000 | | -.289 | | .204 |  |
|  | Northern Central Red Sea | | | .215^*^ | | .068 | | 186 | .018 | | .023 | | .408 |  |
|  | Southern Central Red Sea | | | -.101 | | .067 | | 186 | 1.000 | | -.291 | | .088 |  |
|  | Southern Red Sea | | | -.148 | | .088 | | 186 | .947 | | -.398 | | .102 |  |
| Southern Central Red Sea | Gulf of Aqaba | | | .059 | | .067 | | 186 | 1.000 | | -.132 | | .250 |  |
|  | Northern Central Red Sea | | | .317^*^ | | .040 | | 186 | <.001 | | .204 | | .430 |  |
|  | Northern Red Sea | | | .101 | | .067 | | 186 | 1.000 | | -.088 | | .291 |  |
|  | Southern Red Sea | | | -.046 | | .069 | | 186.000 | 1.000 | | -.241 | | .149 |  |
| Southern Red Sea | Gulf of Aqaba | | | .105 | | .088 | | 186.000 | 1.000 | | -.146 | | .356 |  |
|  | Northern Central Red Sea | | | .363^*^ | | .070 | | 186 | <.001 | | .165 | | .561 |  |
|  | Northern Red Sea | | | .148 | | .088 | | 186 | .947 | | -.102 | | .398 |  |
|  | Southern Central Red Sea | | | .046 | | .069 | | 186.000 | 1.000 | | -.149 | | .241 |  |
| Based on estimated marginal means | | | | | | | | | | | | | | |
| *. The mean difference is significant at the .05 level. | | | | | | | | | | | | | | |
| a. Dependent Variable: ACE_ln. | | | | | | | | | | | | | | |
| c. Adjustment for multiple comparisons: Bonferroni. | | | | | | | | | | | | | | |

| **Univariate Tests^a^** | | | |
| --- | --- | --- | --- |
| Numerator df | Denominator df | F | Sig. |
| 4 | 186.000 | 18.226 | <.001 |
| The F tests the effect of Location. This test is based on the linearly independent pairwise comparisons among the estimated marginal means. | | | |
| a. Dependent Variable: ACE_ln. | | | |

**Supplementary Table 7c: Significant interaction for Layer and Location on Shannon Index**

| **Estimates^a^** | | | | | | |
| --- | --- | --- | --- | --- | --- | --- |
| Layer | Location | Mean | Std. Error | df | 95% Confidence Interval | |
|  |  |  |  |  | Lower Bound | Upper Bound |
| Bathypelagic | Gulf of Aqaba | 4.221 | .460 | 194 | 3.314 | 5.127 |
|  | Northern Central Red Sea | 2.849 | .265 | 194 | 2.325 | 3.372 |
|  | Northern Red Sea | 4.706 | .650 | 194 | 3.424 | 5.988 |
|  | Southern Central Red Sea | 4.568 | .265 | 194 | 4.045 | 5.092 |
|  | Southern Red Sea | 4.419 | .650 | 194.000 | 3.137 | 5.701 |
| Epipelagic | Gulf of Aqaba | 4.038 | .375 | 194 | 3.298 | 4.778 |
|  | Northern Central Red Sea | 3.792 | .110 | 194 | 3.575 | 4.008 |
|  | Northern Red Sea | 3.677 | .174 | 194 | 3.334 | 4.020 |
|  | Southern Central Red Sea | 4.212 | .107 | 194 | 4.002 | 4.423 |
|  | Southern Red Sea | 3.744 | .206 | 194.000 | 3.339 | 4.149 |
| Mesopelagic | Gulf of Aqaba | 4.288 | .375 | 194 | 3.548 | 5.028 |
|  | Northern Central Red Sea | 3.630 | .115 | 194 | 3.403 | 3.856 |
|  | Northern Red Sea | 4.085 | .180 | 194 | 3.729 | 4.440 |
|  | Southern Central Red Sea | 4.127 | .107 | 194 | 3.916 | 4.338 |
|  | Southern Red Sea | 3.801 | .217 | 194.000 | 3.373 | 4.228 |
| a. Dependent Variable: Shannon. | | | | | | |

| **Pairwise Comparisons^a^** | | | | | | | | |
| --- | --- | --- | --- | --- | --- | --- | --- | --- |
| Location | (I) Layer | (J) Layer | Mean Difference (I-J) | Std. Error | df | Sig.^c^ | 95% Confidence Interval for Difference^c^ | |
|  |  |  |  |  |  |  | Lower Bound | Upper Bound |
| Gulf of Aqaba | Bathypelagic | Epipelagic | .183 | .593 | 194 | .759 | -.988 | 1.353 |
|  |  | Mesopelagic | -.067 | .593 | 194 | .910 | -1.237 | 1.103 |
|  | Epipelagic | Bathypelagic | -.183 | .593 | 194 | .759 | -1.353 | .988 |
|  |  | Mesopelagic | -.250 | .531 | 194 | .638 | -1.297 | .797 |
|  | Mesopelagic | Bathypelagic | .067 | .593 | 194 | .910 | -1.103 | 1.237 |
|  |  | Epipelagic | .250 | .531 | 194 | .638 | -.797 | 1.297 |
| Northern Central Red Sea | Bathypelagic | Epipelagic | -.943^*^ | .287 | 194 | .001 | -1.509 | -.377 |
|  |  | Mesopelagic | -.781^*^ | .289 | 194 | .008 | -1.351 | -.211 |
|  | Epipelagic | Bathypelagic | .943^*^ | .287 | 194 | .001 | .377 | 1.509 |
|  |  | Mesopelagic | .162 | .159 | 194 | .310 | -.152 | .476 |
|  | Mesopelagic | Bathypelagic | .781^*^ | .289 | 194 | .008 | .211 | 1.351 |
|  |  | Epipelagic | -.162 | .159 | 194 | .310 | -.476 | .152 |
| Northern Red Sea | Bathypelagic | Epipelagic | 1.029 | .673 | 194 | .128 | -.298 | 2.356 |
|  |  | Mesopelagic | .621 | .675 | 194 | .359 | -.710 | 1.951 |
|  | Epipelagic | Bathypelagic | -1.029 | .673 | 194 | .128 | -2.356 | .298 |
|  |  | Mesopelagic | -.408 | .250 | 194 | .105 | -.902 | .086 |
|  | Mesopelagic | Bathypelagic | -.621 | .675 | 194 | .359 | -1.951 | .710 |
|  |  | Epipelagic | .408 | .250 | 194 | .105 | -.086 | .902 |
| Southern Central Red Sea | Bathypelagic | Epipelagic | .356 | .286 | 194 | .215 | -.208 | .920 |
|  |  | Mesopelagic | .441 | .286 | 194 | .125 | -.123 | 1.005 |
|  | Epipelagic | Bathypelagic | -.356 | .286 | 194 | .215 | -.920 | .208 |
|  |  | Mesopelagic | .085 | .151 | 194 | .572 | -.213 | .384 |
|  | Mesopelagic | Bathypelagic | -.441 | .286 | 194 | .125 | -1.005 | .123 |
|  |  | Epipelagic | -.085 | .151 | 194 | .572 | -.384 | .213 |
| Southern Red Sea | Bathypelagic | Epipelagic | .675 | .682 | 194 | .323 | -.669 | 2.020 |
|  |  | Mesopelagic | .618 | .685 | 194 | .368 | -.733 | 1.970 |
|  | Epipelagic | Bathypelagic | -.675 | .682 | 194 | .323 | -2.020 | .669 |
|  |  | Mesopelagic | -.057 | .299 | 194.000 | .849 | -.646 | .532 |
|  | Mesopelagic | Bathypelagic | -.618 | .685 | 194 | .368 | -1.970 | .733 |
|  |  | Epipelagic | .057 | .299 | 194.000 | .849 | -.532 | .646 |
| Based on estimated marginal means | | | | | | | | |
| *. The mean difference is significant at the .05 level. | | | | | | | | |
| a. Dependent Variable: Shannon. | | | | | | | | |
| c. Adjustment for multiple comparisons: Least Significant Difference (equivalent to no adjustments). | | | | | | | | |

| **Univariate Tests^a^** | | | | |
| --- | --- | --- | --- | --- |
| Location | Numerator df | Denominator df | F | Sig. |
| Gulf of Aqaba | 2 | 194.000 | .117 | .890 |
| Northern Central Red Sea | 2 | 194 | 5.402 | .005 |
| Northern Red Sea | 2 | 194 | 2.117 | .123 |
| Southern Central Red Sea | 2 | 194.000 | 1.203 | .303 |
| Southern Red Sea | 2 | 194 | .491 | .613 |
| Each F tests the simple effects of Layer within each level combination of the other effects shown. These tests are based on the linearly independent pairwise comparisons among the estimated marginal means. | | | | |
| a. Dependent Variable: Shannon. | | | | |

**Mixed Model Analysis**

| **Model Dimension^a^** | | | |
| --- | --- | --- | --- |
|  | | Number of Levels | Number of Parameters |
| Fixed Effects | Intercept | 1 | 1 |
|  | Layer | 3 | 2 |
|  | Location | 5 | 4 |
|  | Layer * Location | 15 | 8 |
| Residual | |  | 1 |
| Total | | 24 | 16 |
| a. Dependent Variable: Shannon. | | | |

| **Information Criteria^a^** | |
| --- | --- |
| -2 Restricted Log Likelihood | 413.84558438 |
| Akaike's Information Criterion (AIC) | 415.84558438 |
| Hurvich and Tsai's Criterion (AICC) | 415.86641771 |
| Bozdogan's Criterion (CAIC) | 420.11344254 |
| Schwarz's Bayesian Criterion (BIC) | 419.11344254 |
| The information criteria are displayed in smaller-is-better form. | |
| a. Dependent Variable: Shannon. | |

| **Coefficients of Determination** | | |
| --- | --- | --- |
| Pseudo-R Square Measures | Marginal | .200 |
|  | Conditional | .200 |

**Fixed Effects**

| **Type III Tests of Fixed Effects^a^** | | | | |
| --- | --- | --- | --- | --- |
| Source | Numerator df | Denominator df | F | Sig. |
| Intercept | 1 | 194 | 2155.360 | <.001 |
| Layer | 2 | 194.000 | .661 | .518 |
| Location | 4 | 194 | 9.923 | <.001 |
| Layer * Location | 8 | 194.000 | 2.304 | .022 |
| a. Dependent Variable: Shannon. | | | | |

**Covariance Parameters**

| **Estimates of Covariance Parameters^a^** | | |
| --- | --- | --- |
| Parameter | Estimate | Std. Error |
| Residual | .423 | .043 |
| a. Dependent Variable: Shannon. | | |

**Estimated Marginal Means**

| **Estimates^a^** | | | | | | |
| --- | --- | --- | --- | --- | --- | --- |
| Layer | Location | Mean | Std. Error | df | 95% Confidence Interval | |
|  |  |  |  |  | Lower Bound | Upper Bound |
| Bathypelagic | Gulf of Aqaba | 4.221 | .460 | 194 | 3.314 | 5.127 |
|  | Northern Central Red Sea | 2.849 | .265 | 194 | 2.325 | 3.372 |
|  | Northern Red Sea | 4.706 | .650 | 194 | 3.424 | 5.988 |
|  | Southern Central Red Sea | 4.568 | .265 | 194 | 4.045 | 5.092 |
|  | Southern Red Sea | 4.419 | .650 | 194.000 | 3.137 | 5.701 |
| Epipelagic | Gulf of Aqaba | 4.038 | .375 | 194 | 3.298 | 4.778 |
|  | Northern Central Red Sea | 3.792 | .110 | 194 | 3.575 | 4.008 |
|  | Northern Red Sea | 3.677 | .174 | 194 | 3.334 | 4.020 |
|  | Southern Central Red Sea | 4.212 | .107 | 194 | 4.002 | 4.423 |
|  | Southern Red Sea | 3.744 | .206 | 194.000 | 3.339 | 4.149 |
| Mesopelagic | Gulf of Aqaba | 4.288 | .375 | 194 | 3.548 | 5.028 |
|  | Northern Central Red Sea | 3.630 | .115 | 194 | 3.403 | 3.856 |
|  | Northern Red Sea | 4.085 | .180 | 194 | 3.729 | 4.440 |
|  | Southern Central Red Sea | 4.127 | .107 | 194 | 3.916 | 4.338 |
|  | Southern Red Sea | 3.801 | .217 | 194.000 | 3.373 | 4.228 |
| a. Dependent Variable: Shannon. | | | | | | |

| **Pairwise Comparisons^a^** | | | | | | | | |
| --- | --- | --- | --- | --- | --- | --- | --- | --- |
| Layer | (I) Location | (J) Location | Mean Difference (I-J) | Std. Error | df | Sig.^c^ | 95% Confidence Interval for Difference^c^ | |
|  |  |  |  |  |  |  | Lower Bound | Upper Bound |
| Bathypelagic | Gulf of Aqaba | Northern Central Red Sea | 1.372^*^ | .531 | 194 | .010 | .326 | 2.419 |
|  |  | Northern Red Sea | -.485 | .796 | 194 | .543 | -2.055 | 1.085 |
|  |  | Southern Central Red Sea | -.347 | .531 | 194 | .514 | -1.394 | .699 |
|  |  | Southern Red Sea | -.198 | .796 | 194 | .804 | -1.769 | 1.372 |
|  | Northern Central Red Sea | Gulf of Aqaba | -1.372^*^ | .531 | 194 | .010 | -2.419 | -.326 |
|  |  | Northern Red Sea | -1.857^*^ | .702 | 194 | .009 | -3.242 | -.472 |
|  |  | Southern Central Red Sea | -1.720^*^ | .375 | 194 | <.001 | -2.460 | -.979 |
|  |  | Southern Red Sea | -1.571^*^ | .702 | 194 | .026 | -2.955 | -.186 |
|  | Northern Red Sea | Gulf of Aqaba | .485 | .796 | 194 | .543 | -1.085 | 2.055 |
|  |  | Northern Central Red Sea | 1.857^*^ | .702 | 194 | .009 | .472 | 3.242 |
|  |  | Southern Central Red Sea | .137 | .702 | 194 | .845 | -1.247 | 1.522 |
|  |  | Southern Red Sea | .286 | .919 | 194 | .756 | -1.527 | 2.099 |
|  | Southern Central Red Sea | Gulf of Aqaba | .347 | .531 | 194 | .514 | -.699 | 1.394 |
|  |  | Northern Central Red Sea | 1.720^*^ | .375 | 194 | <.001 | .979 | 2.460 |
|  |  | Northern Red Sea | -.137 | .702 | 194 | .845 | -1.522 | 1.247 |
|  |  | Southern Red Sea | .149 | .702 | 194 | .832 | -1.236 | 1.534 |
|  | Southern Red Sea | Gulf of Aqaba | .198 | .796 | 194 | .804 | -1.372 | 1.769 |
|  |  | Northern Central Red Sea | 1.571^*^ | .702 | 194 | .026 | .186 | 2.955 |
|  |  | Northern Red Sea | -.286 | .919 | 194 | .756 | -2.099 | 1.527 |
|  |  | Southern Central Red Sea | -.149 | .702 | 194 | .832 | -1.534 | 1.236 |
| Epipelagic | Gulf of Aqaba | Northern Central Red Sea | .247 | .391 | 194 | .529 | -.525 | 1.018 |
|  |  | Northern Red Sea | .361 | .414 | 194 | .383 | -.454 | 1.177 |
|  |  | Southern Central Red Sea | -.174 | .390 | 194 | .656 | -.944 | .595 |
|  |  | Southern Red Sea | .294 | .428 | 194 | .492 | -.550 | 1.138 |
|  | Northern Central Red Sea | Gulf of Aqaba | -.247 | .391 | 194 | .529 | -1.018 | .525 |
|  |  | Northern Red Sea | .115 | .206 | 194 | .578 | -.291 | .520 |
|  |  | Southern Central Red Sea | -.421^*^ | .153 | 194 | .007 | -.723 | -.118 |
|  |  | Southern Red Sea | .048 | .233 | 194 | .838 | -.412 | .507 |
|  | Northern Red Sea | Gulf of Aqaba | -.361 | .414 | 194 | .383 | -1.177 | .454 |
|  |  | Northern Central Red Sea | -.115 | .206 | 194 | .578 | -.520 | .291 |
|  |  | Southern Central Red Sea | -.535^*^ | .204 | 194 | .009 | -.938 | -.133 |
|  |  | Southern Red Sea | -.067 | .269 | 194 | .804 | -.598 | .464 |
|  | Southern Central Red Sea | Gulf of Aqaba | .174 | .390 | 194 | .656 | -.595 | .944 |
|  |  | Northern Central Red Sea | .421^*^ | .153 | 194 | .007 | .118 | .723 |
|  |  | Northern Red Sea | .535^*^ | .204 | 194 | .009 | .133 | .938 |
|  |  | Southern Red Sea | .468^*^ | .232 | 194.000 | .045 | .011 | .925 |
|  | Southern Red Sea | Gulf of Aqaba | -.294 | .428 | 194 | .492 | -1.138 | .550 |
|  |  | Northern Central Red Sea | -.048 | .233 | 194 | .838 | -.507 | .412 |
|  |  | Northern Red Sea | .067 | .269 | 194 | .804 | -.464 | .598 |
|  |  | Southern Central Red Sea | -.468^*^ | .232 | 194.000 | .045 | -.925 | -.011 |
| Mesopelagic | Gulf of Aqaba | Northern Central Red Sea | .658 | .392 | 194 | .095 | -.116 | 1.433 |
|  |  | Northern Red Sea | .203 | .416 | 194 | .626 | -.618 | 1.024 |
|  |  | Southern Central Red Sea | .161 | .390 | 194 | .680 | -.609 | .931 |
|  |  | Southern Red Sea | .487 | .433 | 194.000 | .262 | -.367 | 1.342 |
|  | Northern Central Red Sea | Gulf of Aqaba | -.658 | .392 | 194 | .095 | -1.433 | .116 |
|  |  | Northern Red Sea | -.455^*^ | .214 | 194 | .035 | -.877 | -.034 |
|  |  | Southern Central Red Sea | -.497^*^ | .157 | 194 | .002 | -.807 | -.188 |
|  |  | Southern Red Sea | -.171 | .245 | 194 | .486 | -.655 | .313 |
|  | Northern Red Sea | Gulf of Aqaba | -.203 | .416 | 194 | .626 | -1.024 | .618 |
|  |  | Northern Central Red Sea | .455^*^ | .214 | 194 | .035 | .034 | .877 |
|  |  | Southern Central Red Sea | -.042 | .210 | 194 | .841 | -.455 | .371 |
|  |  | Southern Red Sea | .284 | .282 | 194.000 | .315 | -.272 | .840 |
|  | Southern Central Red Sea | Gulf of Aqaba | -.161 | .390 | 194 | .680 | -.931 | .609 |
|  |  | Northern Central Red Sea | .497^*^ | .157 | 194 | .002 | .188 | .807 |
|  |  | Northern Red Sea | .042 | .210 | 194 | .841 | -.371 | .455 |
|  |  | Southern Red Sea | .326 | .242 | 194.000 | .179 | -.150 | .803 |
|  | Southern Red Sea | Gulf of Aqaba | -.487 | .433 | 194.000 | .262 | -1.342 | .367 |
|  |  | Northern Central Red Sea | .171 | .245 | 194 | .486 | -.313 | .655 |
|  |  | Northern Red Sea | -.284 | .282 | 194.000 | .315 | -.840 | .272 |
|  |  | Southern Central Red Sea | -.326 | .242 | 194.000 | .179 | -.803 | .150 |
| Based on estimated marginal means | | | | | | | | |
| *. The mean difference is significant at the .05 level. | | | | | | | | |
| a. Dependent Variable: Shannon. | | | | | | | | |
| c. Adjustment for multiple comparisons: Least Significant Difference (equivalent to no adjustments). | | | | | | | | |

| **Univariate Tests^a^** | | | | |
| --- | --- | --- | --- | --- |
| Layer | Numerator df | Denominator df | F | Sig. |
| Bathypelagic | 4 | 194.000 | 6.170 | <.001 |
| Epipelagic | 4 | 194 | 2.897 | .023 |
| Mesopelagic | 4 | 194.000 | 3.057 | .018 |
| Each F tests the simple effects of Location within each level combination of the other effects shown. These tests are based on the linearly independent pairwise comparisons among the estimated marginal means. | | | | |
| a. Dependent Variable: Shannon. | | | | |

**Supplementary Table 7d: Significant interaction for Layer and Location on Simpson Index**

| **Estimates^a^** | | | | | | |
| --- | --- | --- | --- | --- | --- | --- |
| Layer | Location | Mean | Std. Error | df | 95% Confidence Interval | |
|  |  |  |  |  | Lower Bound | Upper Bound |
| Bathypelagic | Gulf of Aqaba | 1.398 | .018 | 194 | 1.362 | 1.434 |
|  | Northern Central Red Sea | 1.327 | .011 | 194 | 1.306 | 1.348 |
|  | Northern Red Sea | 1.406 | .026 | 194 | 1.354 | 1.457 |
|  | Southern Central Red Sea | 1.404 | .011 | 194 | 1.383 | 1.425 |
|  | Southern Red Sea | 1.399 | .026 | 194.000 | 1.347 | 1.450 |
| Epipelagic | Gulf of Aqaba | 1.398 | .015 | 194 | 1.369 | 1.428 |
|  | Northern Central Red Sea | 1.396 | .004 | 194 | 1.387 | 1.404 |
|  | Northern Red Sea | 1.381 | .007 | 194.000 | 1.368 | 1.395 |
|  | Southern Central Red Sea | 1.393 | .004 | 194.000 | 1.385 | 1.401 |
|  | Southern Red Sea | 1.368 | .008 | 194.000 | 1.351 | 1.384 |
| Mesopelagic | Gulf of Aqaba | 1.400 | .015 | 194 | 1.370 | 1.429 |
|  | Northern Central Red Sea | 1.391 | .005 | 194 | 1.382 | 1.400 |
|  | Northern Red Sea | 1.393 | .007 | 194 | 1.379 | 1.407 |
|  | Southern Central Red Sea | 1.392 | .004 | 194 | 1.384 | 1.401 |
|  | Southern Red Sea | 1.380 | .009 | 194 | 1.363 | 1.397 |
| a. Dependent Variable: Simpson_sqrt. | | | | | | |

| **Pairwise Comparisons^a^** | | | | | | | | |
| --- | --- | --- | --- | --- | --- | --- | --- | --- |
| Location | (I) Layer | (J) Layer | Mean Difference (I-J) | Std. Error | df | Sig.^c^ | 95% Confidence Interval for Difference^c^ | |
|  |  |  |  |  |  |  | Lower Bound | Upper Bound |
| Gulf of Aqaba | Bathypelagic | Epipelagic | .000 | .024 | 194 | .989 | -.047 | .047 |
|  |  | Mesopelagic | -.002 | .024 | 194 | .947 | -.049 | .045 |
|  | Epipelagic | Bathypelagic | .000 | .024 | 194 | .989 | -.047 | .047 |
|  |  | Mesopelagic | -.001 | .021 | 194 | .953 | -.043 | .041 |
|  | Mesopelagic | Bathypelagic | .002 | .024 | 194 | .947 | -.045 | .049 |
|  |  | Epipelagic | .001 | .021 | 194 | .953 | -.041 | .043 |
| Northern Central Red Sea | Bathypelagic | Epipelagic | -.069^*^ | .012 | 194 | <.001 | -.092 | -.046 |
|  |  | Mesopelagic | -.064^*^ | .012 | 194 | <.001 | -.087 | -.041 |
|  | Epipelagic | Bathypelagic | .069^*^ | .012 | 194 | <.001 | .046 | .092 |
|  |  | Mesopelagic | .005 | .006 | 194 | .426 | -.008 | .018 |
|  | Mesopelagic | Bathypelagic | .064^*^ | .012 | 194 | <.001 | .041 | .087 |
|  |  | Epipelagic | -.005 | .006 | 194 | .426 | -.018 | .008 |
| Northern Red Sea | Bathypelagic | Epipelagic | .024 | .027 | 194 | .370 | -.029 | .078 |
|  |  | Mesopelagic | .013 | .027 | 194 | .639 | -.041 | .066 |
|  | Epipelagic | Bathypelagic | -.024 | .027 | 194 | .370 | -.078 | .029 |
|  |  | Mesopelagic | -.012 | .010 | 194 | .252 | -.031 | .008 |
|  | Mesopelagic | Bathypelagic | -.013 | .027 | 194 | .639 | -.066 | .041 |
|  |  | Epipelagic | .012 | .010 | 194 | .252 | -.008 | .031 |
| Southern Central Red Sea | Bathypelagic | Epipelagic | .011 | .011 | 194 | .324 | -.011 | .034 |
|  |  | Mesopelagic | .012 | .011 | 194 | .295 | -.011 | .035 |
|  | Epipelagic | Bathypelagic | -.011 | .011 | 194 | .324 | -.034 | .011 |
|  |  | Mesopelagic | .001 | .006 | 194 | .909 | -.011 | .013 |
|  | Mesopelagic | Bathypelagic | -.012 | .011 | 194 | .295 | -.035 | .011 |
|  |  | Epipelagic | -.001 | .006 | 194 | .909 | -.013 | .011 |
| Southern Red Sea | Bathypelagic | Epipelagic | .031 | .027 | 194 | .256 | -.023 | .085 |
|  |  | Mesopelagic | .019 | .028 | 194 | .499 | -.036 | .073 |
|  | Epipelagic | Bathypelagic | -.031 | .027 | 194 | .256 | -.085 | .023 |
|  |  | Mesopelagic | -.013 | .012 | 194.000 | .297 | -.036 | .011 |
|  | Mesopelagic | Bathypelagic | -.019 | .028 | 194 | .499 | -.073 | .036 |
|  |  | Epipelagic | .013 | .012 | 194.000 | .297 | -.011 | .036 |
| Based on estimated marginal means | | | | | | | | |
| *. The mean difference is significant at the .05 level. | | | | | | | | |
| a. Dependent Variable: Simpson_sqrt. | | | | | | | | |
| c. Adjustment for multiple comparisons: Least Significant Difference (equivalent to no adjustments). | | | | | | | | |

| **Univariate Tests^a^** | | | | |
| --- | --- | --- | --- | --- |
| Location | Numerator df | Denominator df | F | Sig. |
| Gulf of Aqaba | 2 | 194.000 | .003 | .997 |
| Northern Central Red Sea | 2 | 194.000 | 18.277 | <.001 |
| Northern Red Sea | 2 | 194 | .908 | .405 |
| Southern Central Red Sea | 2 | 194.000 | .566 | .569 |
| Southern Red Sea | 2 | 194 | .990 | .374 |
| Each F tests the simple effects of Layer within each level combination of the other effects shown. These tests are based on the linearly independent pairwise comparisons among the estimated marginal means. | | | | |
| a. Dependent Variable: Simpson_sqrt. | | | | |

**Mixed Model Analysis**

| **Model Dimension^a^** | | | |
| --- | --- | --- | --- |
|  | | Number of Levels | Number of Parameters |
| Fixed Effects | Intercept | 1 | 1 |
|  | Layer | 3 | 2 |
|  | Location | 5 | 4 |
|  | Layer * Location | 15 | 8 |
| Residual | |  | 1 |
| Total | | 24 | 16 |
| a. Dependent Variable: Simpson_sqrt. | | | |

| **Information Criteria^a^** | |
| --- | --- |
| -2 Restricted Log Likelihood | -833.48865209 |
| Akaike's Information Criterion (AIC) | -831.48865209 |
| Hurvich and Tsai's Criterion (AICC) | -831.46781876 |
| Bozdogan's Criterion (CAIC) | -827.22079393 |
| Schwarz's Bayesian Criterion (BIC) | -828.22079393 |
| The information criteria are displayed in smaller-is-better form. | |
| a. Dependent Variable: Simpson_sqrt. | |

| **Coefficients of Determination** | | |
| --- | --- | --- |
| Pseudo-R Square Measures | Marginal | .209 |
|  | Conditional | .209 |

**Fixed Effects**

| **Type III Tests of Fixed Effects^a^** | | | | |
| --- | --- | --- | --- | --- |
| Source | Numerator df | Denominator df | F | Sig. |
| Intercept | 1 | 194 | 160124.199 | <.001 |
| Layer | 2 | 194.000 | .281 | .755 |
| Location | 4 | 194 | 5.611 | <.001 |
| Layer * Location | 8 | 194.000 | 4.314 | <.001 |
| a. Dependent Variable: Simpson_sqrt. | | | | |

**Covariance Parameters**

| **Estimates of Covariance Parameters^a^** | | |
| --- | --- | --- |
| Parameter | Estimate | Std. Error |
| Residual | .001 | 6.921E-5 |
| a. Dependent Variable: Simpson_sqrt. | | |

**Estimated Marginal Means**

| **Estimates^a^** | | | | | | |
| --- | --- | --- | --- | --- | --- | --- |
| Layer | Location | Mean | Std. Error | df | 95% Confidence Interval | |
|  |  |  |  |  | Lower Bound | Upper Bound |
| Bathypelagic | Gulf of Aqaba | 1.398 | .018 | 194 | 1.362 | 1.434 |
|  | Northern Central Red Sea | 1.327 | .011 | 194 | 1.306 | 1.348 |
|  | Northern Red Sea | 1.406 | .026 | 194 | 1.354 | 1.457 |
|  | Southern Central Red Sea | 1.404 | .011 | 194 | 1.383 | 1.425 |
|  | Southern Red Sea | 1.399 | .026 | 194.000 | 1.347 | 1.450 |
| Epipelagic | Gulf of Aqaba | 1.398 | .015 | 194 | 1.369 | 1.428 |
|  | Northern Central Red Sea | 1.396 | .004 | 194 | 1.387 | 1.404 |
|  | Northern Red Sea | 1.381 | .007 | 194.000 | 1.368 | 1.395 |
|  | Southern Central Red Sea | 1.393 | .004 | 194.000 | 1.385 | 1.401 |
|  | Southern Red Sea | 1.368 | .008 | 194.000 | 1.351 | 1.384 |
| Mesopelagic | Gulf of Aqaba | 1.400 | .015 | 194 | 1.370 | 1.429 |
|  | Northern Central Red Sea | 1.391 | .005 | 194 | 1.382 | 1.400 |
|  | Northern Red Sea | 1.393 | .007 | 194 | 1.379 | 1.407 |
|  | Southern Central Red Sea | 1.392 | .004 | 194 | 1.384 | 1.401 |
|  | Southern Red Sea | 1.380 | .009 | 194 | 1.363 | 1.397 |
| a. Dependent Variable: Simpson_sqrt. | | | | | | |

| **Pairwise Comparisons^a^** | | | | | | | | | |
| --- | --- | --- | --- | --- | --- | --- | --- | --- | --- |
| Layer | (I) Location | (J) Location | Mean Difference (I-J) | Std. Error | df | Sig.^c^ | 95% Confidence Interval for Difference^c^ | |  |
|  |  |  |  |  |  |  | Lower Bound | Upper Bound |  |
| Bathypelagic | Gulf of Aqaba | Northern Central Red Sea | .071^*^ | .021 | 194 | <.001 | .029 | .113 |  |
|  |  | Northern Red Sea | -.008 | .032 | 194 | .808 | -.071 | .055 |  |
|  |  | Southern Central Red Sea | -.006 | .021 | 194 | .763 | -.048 | .036 |  |
|  |  | Southern Red Sea | -.001 | .032 | 194 | .982 | -.064 | .062 |  |
|  | Northern Central Red Sea | Gulf of Aqaba | -.071^*^ | .021 | 194 | <.001 | -.113 | -.029 |  |
|  |  | Northern Red Sea | -.079^*^ | .028 | 194 | .006 | -.135 | -.024 |  |
|  |  | Southern Central Red Sea | -.078^*^ | .015 | 194 | <.001 | -.108 | -.048 |  |
|  |  | Southern Red Sea | -.072^*^ | .028 | 194 | .011 | -.128 | -.016 |  |
|  | Northern Red Sea | Gulf of Aqaba | .008 | .032 | 194 | .808 | -.055 | .071 |  |
|  |  | Northern Central Red Sea | .079^*^ | .028 | 194 | .006 | .024 | .135 |  |
|  |  | Southern Central Red Sea | .001 | .028 | 194 | .962 | -.054 | .057 |  |
|  |  | Southern Red Sea | .007 | .037 | 194 | .849 | -.066 | .080 |  |
|  | Southern Central Red Sea | Gulf of Aqaba | .006 | .021 | 194 | .763 | -.036 | .048 |  |
|  |  | Northern Central Red Sea | .078^*^ | .015 | 194 | <.001 | .048 | .108 |  |
|  |  | Northern Red Sea | -.001 | .028 | 194 | .962 | -.057 | .054 |  |
|  |  | Southern Red Sea | .006 | .028 | 194 | .839 | -.050 | .061 |  |
|  | Southern Red Sea | Gulf of Aqaba | .001 | .032 | 194 | .982 | -.062 | .064 |  |
|  |  | Northern Central Red Sea | .072^*^ | .028 | 194 | .011 | .016 | .128 |  |
|  |  | Northern Red Sea | -.007 | .037 | 194 | .849 | -.080 | .066 |  |
|  |  | Southern Central Red Sea | -.006 | .028 | 194 | .839 | -.061 | .050 |  |
| Epipelagic | Gulf of Aqaba | Northern Central Red Sea | .003 | .016 | 194 | .870 | -.028 | .034 |  |
|  |  | Northern Red Sea | .017 | .017 | 194 | .312 | -.016 | .050 |  |
|  |  | Southern Central Red Sea | .005 | .016 | 194 | .738 | -.026 | .036 |  |
|  |  | Southern Red Sea | .031 | .017 | 194 | .075 | -.003 | .065 |  |
|  | Northern Central Red Sea | Gulf of Aqaba | -.003 | .016 | 194 | .870 | -.034 | .028 |  |
|  |  | Northern Red Sea | .014 | .008 | 194 | .086 | -.002 | .031 |  |
|  |  | Southern Central Red Sea | .003 | .006 | 194.000 | .664 | -.009 | .015 |  |
|  |  | Southern Red Sea | .028^*^ | .009 | 194 | .003 | .010 | .047 |  |
|  | Northern Red Sea | Gulf of Aqaba | -.017 | .017 | 194 | .312 | -.050 | .016 |  |
|  |  | Northern Central Red Sea | -.014 | .008 | 194 | .086 | -.031 | .002 |  |
|  |  | Southern Central Red Sea | -.012 | .008 | 194 | .159 | -.028 | .005 |  |
|  |  | Southern Red Sea | .014 | .011 | 194 | .199 | -.007 | .035 |  |
|  | Southern Central Red Sea | Gulf of Aqaba | -.005 | .016 | 194 | .738 | -.036 | .026 |  |
|  |  | Northern Central Red Sea | -.003 | .006 | 194.000 | .664 | -.015 | .009 |  |
|  |  | Northern Red Sea | .012 | .008 | 194 | .159 | -.005 | .028 |  |
|  |  | Southern Red Sea | .026^*^ | .009 | 194.000 | .007 | .007 | .044 |  |
|  | Southern Red Sea | Gulf of Aqaba | -.031 | .017 | 194 | .075 | -.065 | .003 |  |
|  |  | Northern Central Red Sea | -.028^*^ | .009 | 194 | .003 | -.047 | -.010 |  |
|  |  | Northern Red Sea | -.014 | .011 | 194 | .199 | -.035 | .007 |  |
|  |  | Southern Central Red Sea | -.026^*^ | .009 | 194.000 | .007 | -.044 | -.007 |  |
| Mesopelagic | Gulf of Aqaba | Northern Central Red Sea | .009 | .016 | 194 | .572 | -.022 | .040 |  |
|  |  | Northern Red Sea | .007 | .017 | 194 | .696 | -.026 | .040 |  |
|  |  | Southern Central Red Sea | .007 | .016 | 194 | .646 | -.024 | .038 |  |
|  |  | Southern Red Sea | .020 | .017 | 194 | .264 | -.015 | .054 |  |
|  | Northern Central Red Sea | Gulf of Aqaba | -.009 | .016 | 194 | .572 | -.040 | .022 |  |
|  |  | Northern Red Sea | -.002 | .009 | 194 | .782 | -.019 | .015 |  |
|  |  | Southern Central Red Sea | -.002 | .006 | 194 | .785 | -.014 | .011 |  |
|  |  | Southern Red Sea | .011 | .010 | 194 | .284 | -.009 | .030 |  |
|  | Northern Red Sea | Gulf of Aqaba | -.007 | .017 | 194 | .696 | -.040 | .026 |  |
|  |  | Northern Central Red Sea | .002 | .009 | 194 | .782 | -.015 | .019 |  |
|  |  | Southern Central Red Sea | .001 | .008 | 194 | .938 | -.016 | .017 |  |
|  |  | Southern Red Sea | .013 | .011 | 194 | .254 | -.009 | .035 |  |
|  | Southern Central Red Sea | Gulf of Aqaba | -.007 | .016 | 194 | .646 | -.038 | .024 |  |
|  |  | Northern Central Red Sea | .002 | .006 | 194 | .785 | -.011 | .014 |  |
|  |  | Northern Red Sea | -.001 | .008 | 194 | .938 | -.017 | .016 |  |
|  |  | Southern Red Sea | .012 | .010 | 194 | .206 | -.007 | .031 |  |
|  | Southern Red Sea | Gulf of Aqaba | -.020 | .017 | 194 | .264 | -.054 | .015 |  |
|  |  | Northern Central Red Sea | -.011 | .010 | 194 | .284 | -.030 | .009 |  |
|  |  | Northern Red Sea | -.013 | .011 | 194 | .254 | -.035 | .009 |  |
|  |  | Southern Central Red Sea | -.012 | .010 | 194 | .206 | -.031 | .007 |  |
| Based on estimated marginal means | | | | | | | | | |
| *. The mean difference is significant at the .05 level. | | | | | | | | | |
| a. Dependent Variable: Simpson_sqrt. | | | | | | | | | |
| c. Adjustment for multiple comparisons: Least Significant Difference (equivalent to no adjustments). | | | | | | | | | |

| **Univariate Tests^a^** | | | | |
| --- | --- | --- | --- | --- |
| Layer | Numerator df | Denominator df | F | Sig. |
| Bathypelagic | 4 | 194.000 | 7.993 | <.001 |
| Epipelagic | 4 | 194.000 | 2.850 | .025 |
| Mesopelagic | 4 | 194 | .522 | .720 |
| Each F tests the simple effects of Location within each level combination of the other effects shown. These tests are based on the linearly independent pairwise comparisons among the estimated marginal means. | | | | |
| a. Dependent Variable: Simpson_sqrt. | | | | |

**Supplementary Table 7e: Significant effect of Location on Fisher Index**

| **Estimates^a^** | | | | | |
| --- | --- | --- | --- | --- | --- |
| Location | Mean | Std. Error | df | 95% Confidence Interval | |
|  |  |  |  | Lower Bound | Upper Bound |
| Gulf of Aqaba | 4.397 | .047 | 194 | 4.305 | 4.489 |
| Northern Central Red Sea | 4.212 | .021 | 194 | 4.171 | 4.252 |
| Northern Red Sea | 4.388 | .046 | 194 | 4.297 | 4.479 |
| Southern Central Red Sea | 4.471 | .020 | 194 | 4.431 | 4.511 |
| Southern Red Sea | 4.479 | .048 | 194.000 | 4.385 | 4.573 |
|  | | | | | |

| **Pairwise Comparisons^a^** | | | | | | | | | | | |
| --- | --- | --- | --- | --- | --- | --- | --- | --- | --- | --- | --- |
| (I) Location | | (J) Location | | Mean Difference (I-J) | | Std. Error | | df | Sig.^c^ | 95% Confidence Interval for Difference^c^ | |
|  |  |  |  |  |  |  |  |  |  | Lower Bound | Upper Bound |
| Gulf of Aqaba | | Northern Central Red Sea | | .185^*^ | | .051 | | 194 | .004 | .040 | .330 |
|  |  | Northern Red Sea | | .009 | | .066 | | 194 | 1.000 | -.178 | .195 |
|  |  | Southern Central Red Sea | | -.074 | | .051 | | 194 | 1.000 | -.219 | .070 |
|  |  | Southern Red Sea | | -.082 | | .067 | | 194 | 1.000 | -.272 | .107 |
| Northern Central Red Sea | | Gulf of Aqaba | | -.185^*^ | | .051 | | 194 | .004 | -.330 | -.040 |
|  |  | Northern Red Sea | | -.176^*^ | | .051 | | 194 | .006 | -.320 | -.032 |
|  |  | Southern Central Red Sea | | -.259^*^ | | .029 | | 194 | <.001 | -.341 | -.177 |
|  |  | Southern Red Sea | | -.267^*^ | | .052 | | 194 | <.001 | -.414 | -.120 |
| Northern Red Sea | | Gulf of Aqaba | | -.009 | | .066 | | 194 | 1.000 | -.195 | .178 |
|  |  | Northern Central Red Sea | | .176^*^ | | .051 | | 194 | .006 | .032 | .320 |
|  |  | Southern Central Red Sea | | -.083 | | .051 | | 194 | 1.000 | -.227 | .060 |
|  |  | Southern Red Sea | | -.091 | | .066 | | 194 | 1.000 | -.279 | .097 |
| Southern Central Red Sea | | Gulf of Aqaba | | .074 | | .051 | | 194 | 1.000 | -.070 | .219 |
|  |  | Northern Central Red Sea | | .259^*^ | | .029 | | 194 | <.001 | .177 | .341 |
|  |  | Northern Red Sea | | .083 | | .051 | | 194 | 1.000 | -.060 | .227 |
|  |  | Southern Red Sea | | -.008 | | .052 | | 194 | 1.000 | -.155 | .139 |
| Southern Red Sea | | Gulf of Aqaba | | .082 | | .067 | | 194 | 1.000 | -.107 | .272 |
|  |  | Northern Central Red Sea | | .267^*^ | | .052 | | 194 | <.001 | .120 | .414 |
|  |  | Northern Red Sea | | .091 | | .066 | | 194 | 1.000 | -.097 | .279 |
|  |  | Southern Central Red Sea | | .008 | | .052 | | 194 | 1.000 | -.139 | .155 |
| Based on estimated marginal means | | | | | | | | | | | |
| *. The mean difference is significant at the .05 level. | | | | | | | | | | | |
| a. Dependent Variable: Fisher_lnxa. | | | | | | | | | | | |
| c. Adjustment for multiple comparisons: Bonferroni. | | | | | | | | | | | |
| **Univariate Tests^a^** | | | | | | |  |  |  |  |  |
| Numerator df | Denominator df | | F | | Sig. | |  |  |  |  |  |
| 4 | 194.000 | | 22.317 | | <.001 | |  |  |  |  |  |
| The F tests the effect of Location. This test is based on the linearly independent pairwise comparisons among the estimated marginal means. | | | | | | |  |  |  |  |  |
| a. Dependent Variable: Fisher_lnxa. | | | | | | |  |  |  |  |  |
