# Supplementary figures and images for: Particle‐Associated Bacterioplankton Communities Across the Red Sea
Source: Environ Microbiol. 2025 Mar 17;27(3):e70075. doi: 10.1111/1462-2920.70075 (PMC11914372; doi:10.1111/1462-2920.70075)

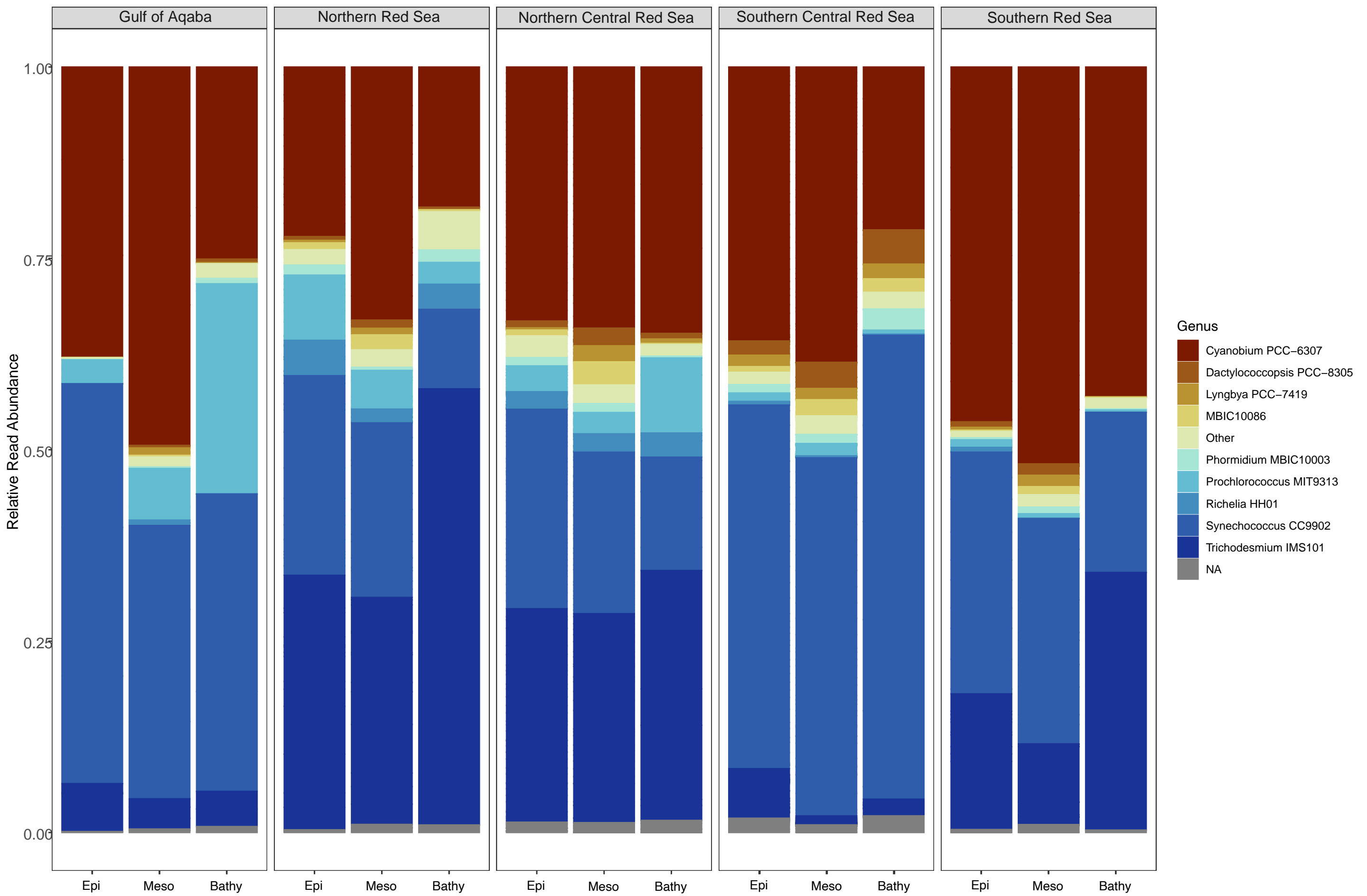

Supplement: Supplementary file 10 — File S10. Relative abundance plot of Cyanobacteria. [file EMI-27-e70075-s006.pdf]

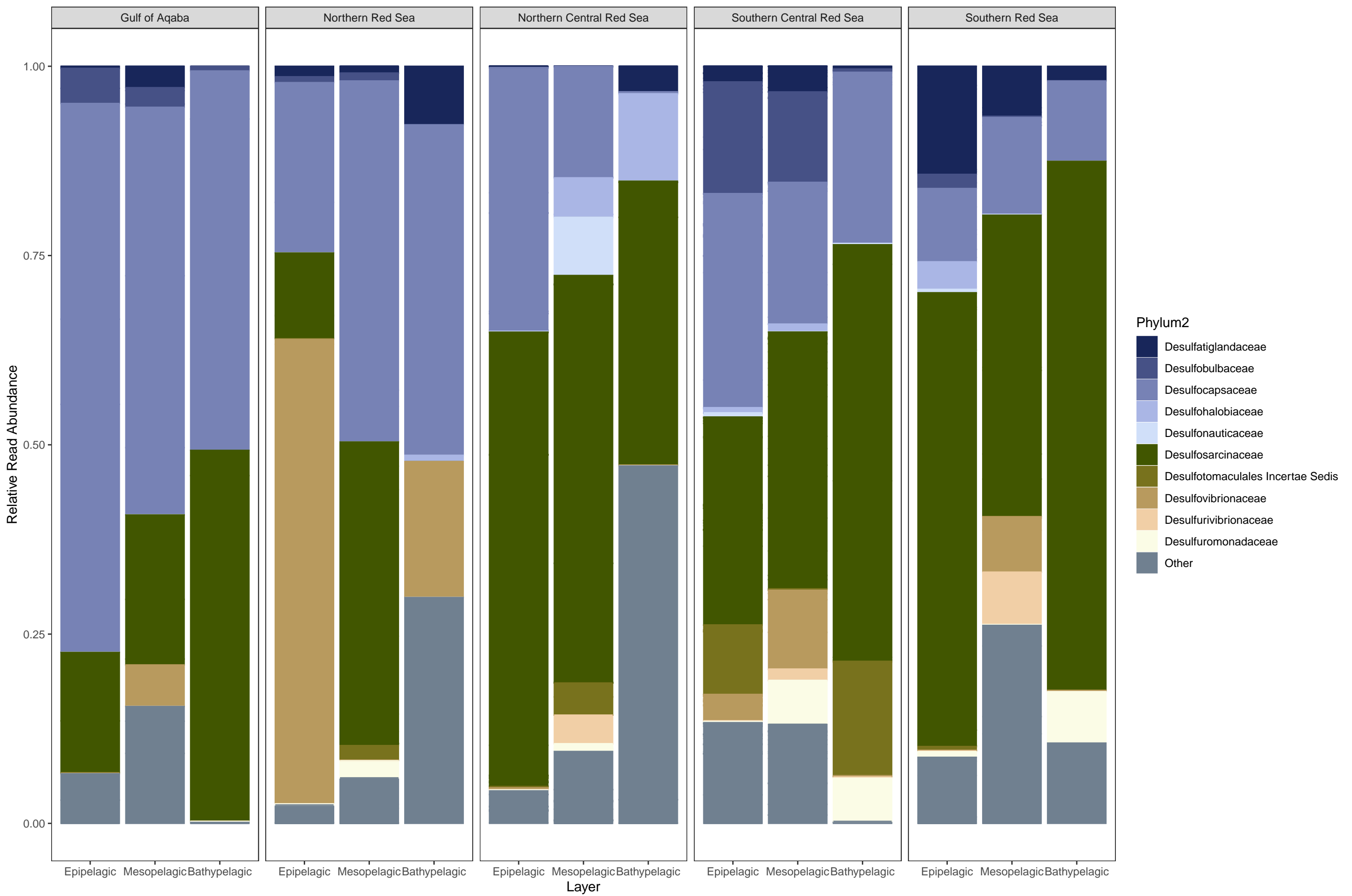

Supplement: Supplementary file 11 — File S11. Relative abundance plot of SRBs. [file EMI-27-e70075-s003.pdf]
